# Abstracts of the 10^th^ International Conference on Cachexia, Sarcopenia and Muscle Wasting, Rome, Italy, 8‐10 December 2017 (Part 2)

**DOI:** 10.1002/jcsm.12284

**Published:** 2018-02-08

**1–07**

**Comparison of sarcopenia and cachexia in men with chronic heart failure: results from the studies investigating co‐morbidities aggravating heart failure (SICA‐HF)**

Amir Emami*^1^, Masakazu Saitoh^1^, Miroslava Valentova^1^, Anja Sandek^1^, Ruben Evertz^1^, Nicole Ebner^1^, Goran Loncar^2,3^, Jochen Springer^1^, Wolfram Doehner^4,8^, Mitja Lainscak^5,6^, Gerd Hasenfuß^1^, Stefan D. Anker^1,7,8^ and Stephan von Haehling^1^

^1^ *Department of Cardiology and Pneumology, University of Göttingen Medical Center, Göttingen, Germany;* ^2^ *Cardiology Department, Clinical Hospital Zvezdara, Belgrade, Serbia;* ^3^ *Faculty of Medicine, University of Belgrade, Belgrade, Serbia;* ^4^ *Center for Stroke Research Berlin, Charite University Medical School, Germany;* ^5^ *Division of Cardiology, General Hospital Murska Sobota, Slovenia;* ^6^ *Faculty of Medicine, University of Ljubljana, Slovenia;* ^7^ *Division of Cardiology and Metabolism – Heart Failure, Cachexia & Sarcopenia, Department of Cardiology, Campus Virchow‐Klinikum, Charité ‐ Universitätsmedizin Berlin, Berlin, Germany;* ^8^ *Berlin‐Brandenburg Center for Regenerative Therapies (BCRT), Charité ‐ Universitätsmedizin Berlin, Berlin, Germany*

**Objectives:** The aim of the present study was to compare the functional impact of changes in body composition as exemplified by heart failure (HF) patients who have cachexia and/or sarcopenia with patients without body wasting.

**Methods:** We prospectively enrolled 207 ambulatory male patients with clinically stable chronic HF who were subgrouped into four groups; (I) patients with sarcopenia without cachexia (sarcopenic HF group), (II) patients with cachexia without sarcopenia (cachectic HF group), (III) patients with cachexia and sarcopenia (sarcopenic cachexia group), and (IV) patients with neither type of wasting (no wasting group).

**Results:** Cachexia was present in 39 (18.8%) of 207 Patients, 14 of whom also fulfilled the characteristics of sarcopenia (sarcopenic cachexia group, 6.7%), whereas 25 did not (cachectic HF group, 12.1%). Sarcopenia without cachexia was present in 30 patients (sarcopenic HF group, 14.4%).

Handgrip strength, peak VO_2_, distance walked in the 6‐MWT, SPPB score, and EQ‐5D index score results were lowest in the sarcopenic cachexia group, with significant differences compared to the no wasting group (all *p* < 0.05). Besides, the sarcopenic cachexia group had the lowest values in quadriceps strength in comparison to the other three groups (all *p* < 0.05). Likewise, the sarcopenic HF group showed lower handgrip strength, quadriceps strength, 6MWT, peak VO_2_, SPPB score and EQ‐5D index score results, with significant differences compared to the no wasting group (all *p* < 0.05). Haemoglobin and IL‐6 levels were significantly reduced in the sarcopenic cachexia group, as compared with the no wasting group (both *p* < 0.05).

**Conclusions:** Losing muscle with or without weight loss appears to have a more pronounced role than weight loss alone, in decreasing functional capacity and QOL among male patients with chronic HF.

**1–10**

**Serial changes in fat, muscle, and bone mass in patients with repeat hospitalization due to worsening heart failure**

Masaaki Konishi*, Eiichi Akiyama, Yasushi Matsuzawa, Nobuhiko Maejima, Noriaki Iwahashi, Kiyoshi Hibi, Masami Kosuge, Kazuo Kimura and Kouichi Tamura

*Yokohama City University Medical Center, Yokohama, Japan*

**Introduction:** Loss of body weight in patients with heart failure (HF) is known as cachexia and associated with poor prognosis. However, there is a paucity of data regarding changes in body composition, namely, fat, muscle, and bone mass.

**Methods:** We retrospectively analysed 32 consecutive patients with heart failure (56% men, left ventricular ejection fraction(LVEF) 41 + −14%, NYHA2.0 + −0.5), who experienced re‐hospitalization due to worsening HF and had two or more results in dual‐energy X‐ray absorptiometry (DXA). To minimize influences by excess fluid in body, DXA was measured at stable state after decongestion therapy.

**Results:** Twenty‐two (69%) patients had an ischemic aetiology and 13 (41%) was categorized as heart failure with preserved LVEF (LVEF > =50). Median interval between the two measurements of DXA was 415 (IQR: 263, 763) days. In the first measurement, low muscle mass defined by the Asian Working Group for Sarcopenia (i.e., <7.0 kg/m2 in men and <5.4 kg/m2 in women) was observed in 72% of patients. During follow‐up period, 69%, 72%, 56%, and 88% of patients experienced weight loss, fat loss, appendicular skeletal muscle loss, and bone loss, defined as any loss of body weight or each body component, respectively. Weight loss was correlated with fat loss (*r* = 0.77, *p* < 0.001) and appendicular skeletal muscle loss (*r* = 0.35, *p* = 0.047), but not with bone loss (*r* = 0.08, *p* = 0.67). Eleven patients with longer history of HF (>5 years) showed greater weight loss than 21 patients with shorter history (−6.5 + −5.7 vs −0.4 + −8.0%, *p* = 0.03) whereas none of age, LVEF, and biomarkers (serum albumin, creatinine, lymphocyte count, C‐reacting protein, B‐type natriuretic peptide) could not predict weight loss, fat loss, and skeletal muscle loss.

**Conclusions:** Weight loss was frequently observed in patients with heart failure requiring repeat hospitalization. Gain and loss of body weight was strongly associated with changes in fat mass in these patients.

**1–12**

**Phase angle measured by bioelectrical impedance analysis in monitoring body composition changes related to treatment and to parenteral nutrition in patients irradiated due of HNC**

Teresa Małecka‐Massalska*^1^, Paweł Gołębiowski^2^, Radosław Mlak^1^, Tomasz Powrózek^1^, Monika Prendecka^1^ and Anna Brzozowska^2^ and Maria Mazurkiewicz

^1^ *Department of Human Physiology, Medical University of Lublin, Radziwiłłowska 11 Street20–080, Lublin, Poland;* ^2^ *Department of Oncology, Medical University of Lublin, Jaczewskiego 7 Street20–090, Lublin, Poland*

**Introduction:** Every year, over 600 thousands new cases of Head and Neck Cancer (HNC) are diagnosed. Progressive weight loss and malnutrition are generally found in those patients, especially under treatment (surgery, radiotherapy [RTH], chemotherapy [CTH]) conditions. Nutritional deficiencies have a significant influence on mortality and quality of life in patients with HNC.

Bioelectrical impedance phase angle (PA) obtained by bioelectrical impedance analysis (BIA), as a measurable indicator of the body condition at the cellular level, is an alternative parameter to laboratory and anthropometric methods to measure and monitor nutritional status of patients with cancer. The aim of the study was to monitor the changes of PA in adult patients with HNC before each RTH cycle classified as well‐nourished, moderately malnourished and severely malnourished according to the SGA scale and the effect of nutritional intervention on the value of PA.

**Methods:** HNC patients were included in the study (*n* = 30, men, stages: I‐IV). All participants were irradiated using IMRT technique (doses: 50‐70Gy). Baseline assessment included: demographic, tumour related, nutritional and clinical evaluation as well as laboratory tests (albumin, prealbumin, transferrin, total protein), subjective global assessment (SGA) and PA measured by BIA at 4 frequencies (5/50/100/200 kHz) before each RTH cycle.

**Results:** Significantly higher values of PA (50 kHz) have been found in SGA C patients when compared to those SGA A or B, before 6‐th (5.48 vs 4.45; *p* = 0.0418) and 7‐th cycle of RTH (5.56 vs 4.37; *p* = 0.0095). In patients treated with parenteral nutrition significantly higher values of PA (50 kHz) measured before 4‐th (5.72 vs 4.25; *p* = 0.0124) and 5‐th (5.48 vs 4.38; *p* = 0.0411) cycle of RTH were found.

**Conclusions:** Our results have shown potential usefulness of PA measured by BIA in monitoring body composition changes related to treatment and to parenteral nutrition in patients irradiated due of HNC.

**2–06**

**Anti‐sarcopenic effect suggested by combination of ARB and statin in patients with cardiovascular disease**

Haruhito Harada*, Ryo Shibata, Kazunori Yamaji, Hiroshi Niiyama, Atsushi Katoh and Hisashi Kai

*Department of Cardiology, Kurume University Medical Center, Kurume, Japan*

**Introduction:** Reduction of skeletal muscle mass is the most important component on diagnosis of sarcopenia. Aging and chronic heart failure due to cardiovascular diseases (CVDs) accelerates reduction of skeletal muscle. We previously reported the possibility of statin to treat sarcopenia with CVD. On the other hand, some angiotensin receptor blockers (ARBs), such as telmisartan and irbesartan, induce the activation of PPARγ and the increase of adiponectin, which are known to promote muscle performance and protect skeletal muscle against inflammation and injury. The purpose of this study was to assess the effectiveness of statin and ARB for an anti‐sarcopenic effect in patients with CVD.

**Methods:** Study design was a single center retrospective cross‐sectional analysis. 670 in‐patients with CVD were divided into four groups including patients taking neither stain nor ARB (control), statin alone, ARB alone and both ARB and stain (ARB/statin) for more than 4 weeks. Skeletal muscle volume was assessed by bioelectrical impedance assay. Skeletal muscle index (SMI) and other variables were statistically compared among four groups.

**Results:** No significance in SMI was found in statin and ARB groups compared with control group. However, SMI was significantly higher in ARB/statin group. Sex difference, age, blood pressure, serum albumin and eGFR did not differ among the four groups. On subanalysis of ARB group, the monotherapy with the 2^nd^ generation ARB (telmisartan, olmesartan, irbesartan and azilsartan), but not with the 1^st^ generation ARB (losartan, valsartan and candesartan), significantly increased SMI. However, SMI was increased significantly by the 1^st^ generation ARBs when used concurrently with a statin.

**Conclusions:** It is suggested that combination of ARB and statin would be a potential anti‐sarcopenic therapy in patients with CVD.

**2–07**

**Phase angle as a predictor of strength independent of lean mass**

Brianna Bourgeois*^1,2^, Neil Johannsen^1,2^, John Shepherd^3^, Maria Cristina Gonzalez^4^ and Steven B. Heymsfield^1,2^

^1^ *Pennington Biomedical Research Center, Baton Rouge;* ^2^ *Louisiana State University, Baton Rouge, LA;* ^3^ *University of California, San Francisco;* ^4^ *Post‐graduate Program on Health and Behavior, Catholic University of Pelotas, Pelotas, RS, Brazil*

**Introduction:** Dual‐energy x‐ray absorptiometry (DXA) is useful tool for quantifying the amount of skeletal muscle in a person's body; however, the correlation between DXA lean soft tissue mass, a muscle surrogate, and strength is not very strong, making it difficult to predict sarcopenia or cachexia risk from DXA alone. We hypothesized that the introduction of a second variable that describes muscle quality would increase the correlation between lean mass by DXA and strength.

**Methods:** We tested this hypothesis on 129 participants (74 female) who had undergone DXA, bioimpedance analysis (BIA), and strength testing using a Biodex System 4 (Biodex Medical Systems Inc., Shirley, New York) for leg strength and a handgrip dynamometer (Sammons Preston Rolyan, Nottinghamshire, UK) for arm strength. Regression models were created with isokinetic peak torque or grip strength as the dependent variables.

**Results:** As an independent variable alone, DXA right leg lean soft tissue (LST) mass predicted isokinetic right leg strength by Biodex with an R^2^ = 0.60 (*p* < 0.0001). When adding 50 kHz phase angle from BIA (S10, InBody, South Korea) to the regression model, the R^2^ increased to 0.69 (*p* < 0.0001). Similar results were found for both right and left arm grip strength models (**Table**). Phase angle from other BIA devices including the COZY 930 and X SCAN PLUS 970 (Selves Healthcare, Seoul, South Korea) increased the R^2^ values for leg strength to 0.70 and 0.66 (both *p* < 0.0001), respectively. Adding sex and age covariates increased the R^2^ values further but phase angle remained a significant (*p* < 0.01) predictor of strength.

**Conclusions:** The combination of DXA‐measured LST, a muscle mass surrogate, and BIA‐measured phase angle improves upper‐ and lower‐body strength prediction over LST mass alone. These results suggest phase angle can potentially add to a DXA scan protocol for diagnosing sarcopenia and cachexia.

**Table:** 

	**Right Leg Peak Torque (Nm)**		**Left Arm Grip Strength (kg)**		**Right Arm Grip Strength (kg)**	
**Model**	Coefficient	S.E.	Coefficient	S.E.	Coefficient	S.E.
**LST only**						
Intercept	−26.4^*^	12.8	7.0^†^	1.8	6.9^†^	1.8
LST (kg)	20.3^‡^	1.5	8.2^‡^	0.6	7.9^‡^	0.6
R^2^	0.60^‡^		0.60^‡^		0.60^‡^	
**LST + PA**						
Intercept	−113.7^‡^	18.3	−10.0^*^	4.3	−10.7^*^	4.4
LST (kg)	18.1^‡^	1.4	6.2^‡^	0.7	6.0^‡^	0.7
PA (°)	15.9^‡^	2.6	3.8^‡^	0.9	3.8^‡^	0.9
R^2^	0.69^‡^		0.65^‡^		0.66^‡^	

^*^p<0.05, ^†^p<0.01, ^‡^p<0.0001 Abbreviations: PA, phase angle at 50 kHz; S.E., standard error

**2–11**

**A systematic review exploring sources the sources of heterogeneity of the prevalence of sarcopenia in community dwelling older adults**

Alexandra Mayhew*^1^, Krystal Amog^1^, Stuart Phillips^2^, Gianni Parise^2^, Russel de Souza^3^, Lehana Thabane^3^ and Parminder Raina^1^

^1^ *Department of Health Research Methods, Evidence, and Impact, Canadian Longitudinal Study on Aging, McMaster University, Hamilton, Canada;* ^2^ *Department of Kinesiology, McMaster University;* ^3^ *Department of Health Research Methods, Evidence, and Impact, Canadian Longitudinal Study on Aging, McMaster University*

**Background:** Several definitions are used to describe sarcopenia, a progressive decrease in muscle that occurs with aging. Definitions include a measure of muscle mass, and may also include a measure of muscle strength and/or physical function. Each measure can be operationalized using numerous methods and thresholds. The goal of this review was to estimate the prevalence of sarcopenia for each definition in community‐dwelling older adults, and to explore potential sources of heterogeneity observed in studies using the same definition.

**Methods:** A systematic review was conducted searching for sarcopenia and muscle mass terms. Screening and data extraction were conducted in duplicate. Overall prevalence for each sarcopenia definition was estimated using a DerSimonian and Laird's random effect model and stratified by sex and ethnicity. Secondary analyses explored potential sources of heterogeneity within definitions including participant age, muscle mass measurement techniques, and thresholds for muscle mass and gait speed.

**Results:** Using data from the 109 included articles, the lowest pooled prevalence (%, 95% confidence interval) estimates were for the European Working Group on Sarcopenia/Asian Working Group on Sarcopenia (12.9%, 9.9–15.9), International Working Group on Sarcopenia (9.9%, 3.2–16.6), and Foundation for the National Institutes of Health (18.6%, 11.8–25.5) definitions. The highest prevalence estimates were for the appendicular skeletal muscle (ASM)/weight (40.4%, 19.5–61.2), ASM/height (30.4%, 20.4–40.3), ASM regressed on height and weight (30.4%, 20.4–40.3), and appendicular lean mass / body mass index (24.2%, 18.3–30.1) definitions.

**Conclusions:** The prevalence of sarcopenia ranged between 9.9% and 40.4%, depending on the definition**,** suggesting that the definitions may not be measuring the same underlying construct. Within definitions, participant age and the muscle mass cut points may potentially explain differences in prevalence. Overall, this review suggests that there is significant heterogeneity between and within sarcopenia definitions that needs to be better understood prior to using sarcopenia in a clinical context.

**2–32**

**Sarcopenia and sarcopenic obesity in women with and without breast cancer, Brazil**

Larissa Vaz Gonçalves^1,2^, Raquel Machado Schincaglia^1^, Jordana Carolina Godinho Mota^1,2^, Karine Anusca Martins^2^, Jõao Felipe Mota^1^, Ana Luiza Lima Sousa^1^ and Ruffo de Freitas‐Júnior^1,2^

^1^ *Programa de pós‐graduação em Ciência da Saúde, Faculdade de Medicina, Universidade Federal de Goiás, Brazil;* ^2^ *Centro de Referência e Diagnóstico da Mama, CORA, Hospital das Clínicas, Universidade Federal de Goiás, Brazil*

**Objective:** To estimate the prevalence of sarcopenia and sarcopenic obesity in women with and without breast cancer.

**Methods:** A transversal with group control study carried out in a reference center for diagnosis and treatment for breast cancer. Classification of stages of sarcopenia pre‐sarcopenia, relative skeletal muscle index (RSMI) <5.45 kg/m^2^; Sarcopenia, RSMI <5.45 kg/m^2^ associated with manual grip <20 kg (dynamometry) or walking speed <0.8 m/s; And severe sarcopenia, RSMI <5.45 kg/m^2^ associated with manual grip <20 kg and walking speed <0.8 m/s. The diagnosis of sarcopenic obesity was confirmed in those with sarcopenia or severe sarcopenia concomitant with the percentage of total fat mass (DXA‐beam absorptiometry method) greater than 38%. For each menopausal *status* (pre / post‐menopausal), two groups (case‐cancer and control‐without cancer) were matched by age (30 to 80 years).

**Results:** A total of 262 women participated in the study, of which 46.6% were premenopausal women. The mean age of participants was 51.4 years (± 11.3); The premenopausal group was younger (*p* < 0.001). The prevalence of sarcopenia was 5% (*n* = 13), similar between *status* and also between groups; When pre‐sarcopenia was added (*n* = 13), the prevalence was 9.9%. The prevalence of sarcopenic obesity was 4.2% (*n* = 11) in the total sample. In both menopausal *status*, there was an association between sarcopenia and sarcopenic obesity (premenopausal = 100.0% and postmenopausal = 83.3%), with no significant difference when comparing this prevalence between the statuses or between the groups.

**Conclusions:** The prevalence of pre‐sarcopenia was equal to the prevalence of sarcopenia and there was no association with menopausal status or even with the presence of the disease. Obesity‐related sarcopenia was associated with the presence of sarcopenia in both groups and among status. The menopausal status and presence of breast cancer were not associated with the analysed variables.

**2–53**

**High muscle mass associated with reduced prognosis among patients with acute calculous cholecystitis**

Eyal Leibovitz*^1^, Nadav Ben‐David^2^, Lea Shibanov^2^, Sorin Elias^3^ and Mordechai Shimonov^2,4^

^1^ *Department of internal medicine “A”, Yoseftal hospital;* ^2^ *Department of Surgery “A” at the Wolfson Medical Center;* ^3^ *Department of radiology at the Wolfson Medical center, Holon;* ^4^ *The Sackler school of medicine, Tel Aviv University, Israel*

**Background:** Sarcopenia and low muscle mass are considered a bad prognostic marker for many patient populations. We studied body composition and hospital prognosis of patients admitted because of acute calculous cholecystitis (ACC).

**Methods:** a retrospective analysis of medical records of patients admitted to surgical units because of ACC between Jan 1st 2010 and December 31st 2014. Included were patients that had a CT scan during the beginning (first 72 hours) of the admission. Body composition was measured using designated software (SliceOmatic, TomoVIsion, Magog, Canada) at the 3rd lumbar vertebra, using pre‐specified Hounsfield unit values. Patients were divided into two groups based on above or below the median skeletal muscle surface area.

**Results:** Included in the analysis were 159 patients (mean age 71.7 ± 15.8, 54.7% males). Mean ASA score was 2.5 ± 0.8, and 50.8% had a moderately severe disease. Average muscle surface area was 122 ± 31 cm2. Average muscle surface area was higher in males (139 ± 27 cm2compared to 101 ± 20 cm2 in females, *p* < 0.001) and in patients under 65 years of age (135 ± 30 cm2 compared to 117 ± 30 cm2 in over 65 years of age adults, *p* = 0.001). After controlling for age, sex, disease severity, ASA score and visceral fat mass, the general linear model showed that high muscle mass was associated with a tendency towards longer hospital stay (12.3 ± 1.5 days compared to 7.8 ± 1.5 days in patients with low muscle mass, *p* = 0.06). 30 day mortality rates were low and prevented analysis studies. After controlling for age, sex, disease severity, ASA score and the Charlson co‐morbidity index, the Cox regression analysis showed that 1‐year survival was better among patients with low muscle mass (OR 8.102, 95% confidence interval 1.141–57.548, *p* = 0.036).

**Conclusions:** Low muscle surface area was associated with a better prognosis in patients with acute calculous cholecystitis.

**2–66**

**Low muscularity by CT definition is prevalent in both cancer and non‐cancer control populations**

Michael I. Ramage*, Aleksandra Staniszewska, Edi Schuepbach and Ronnen Roubenof and Gabriel Oniscu and Stephen J. Wigmore and D.A. Christopher Deans and James A. Ross and Carsten Jacobi and Richard J.E. Skipworth

*Department of Clinical Surgery, Royal Infirmary of Edinburgh, Scotland, UK*

**Introduction:** Identification of cancer cachexia by CT is commonplace, using defined cut‐points to assess low muscularity [LM] (“sarcopenia”), which correlates with poor outcome. We used CT to assess the prevalence of LM in upper GI cancer patients (UGIC) and non‐cancer controls.

**Methods:** Cachexia was defined using the consensus definition (>5% weight loss or >2% weight loss with LM) (Fearon et al. 2011). CT planimetry at L3 was performed on preoperative scans of patients with UGIC (*n* = 89; M:F 62:27; median 68 yrs), vascular/aortic surgery [AAA] (*n* = 647; M:F 510:137; median 73 yrs), and healthy live kidney donors [LDN] (*n* = 208; M:F 111:97; median 50 yrs). CT muscularity was measured using skeletal muscle index (SMI) with following cut points to define LM: <41cm^2^/m^2^ in females; <43cm^2^/m^2^ in BMI < 25 males; <53cm^2^/m^2^ in BMI > 25 males (Martin et al. 2013).

**Results:** Within UGIC group, 53/89(60%) were cachectic, and of these, 26/53(49%) had low skeletal muscle density. Overall prevalence of LM was 47% (mean 46.97cm^2^/m^2^), with high prevalence in high BMI males compared with low BMI males (63% vs 29%: Chi‐squared *p* = 0.009) and females (37%). In comparison, overall LM prevalence was higher in AAA patients (52%;mean 47.67cm^2^/m^2^), and was similar across females, low BMI males and high BMI males (52% vs 50% vs 60%). In LDN (overall LM 38%;mean 46.76cm^2^/m^2^), a different pattern was observed with a high prevalence of LM in females (50%) and a very low prevalence in low BMI males versus high BMI males (9% vs 36%, Chi‐squared *p* = 0.002).

**Conclusions:** CT‐derived muscularity varies significantly between different patient populations. When using cut‐points derived in a cancer population, low muscularity is prevalent even in healthy adults (particularly women), and is more prevalent in vascular patients than cancer. In males of all groups, obesity is associated with LM. Human CT body composition analysis is poorly understood and population‐specific studies are required.

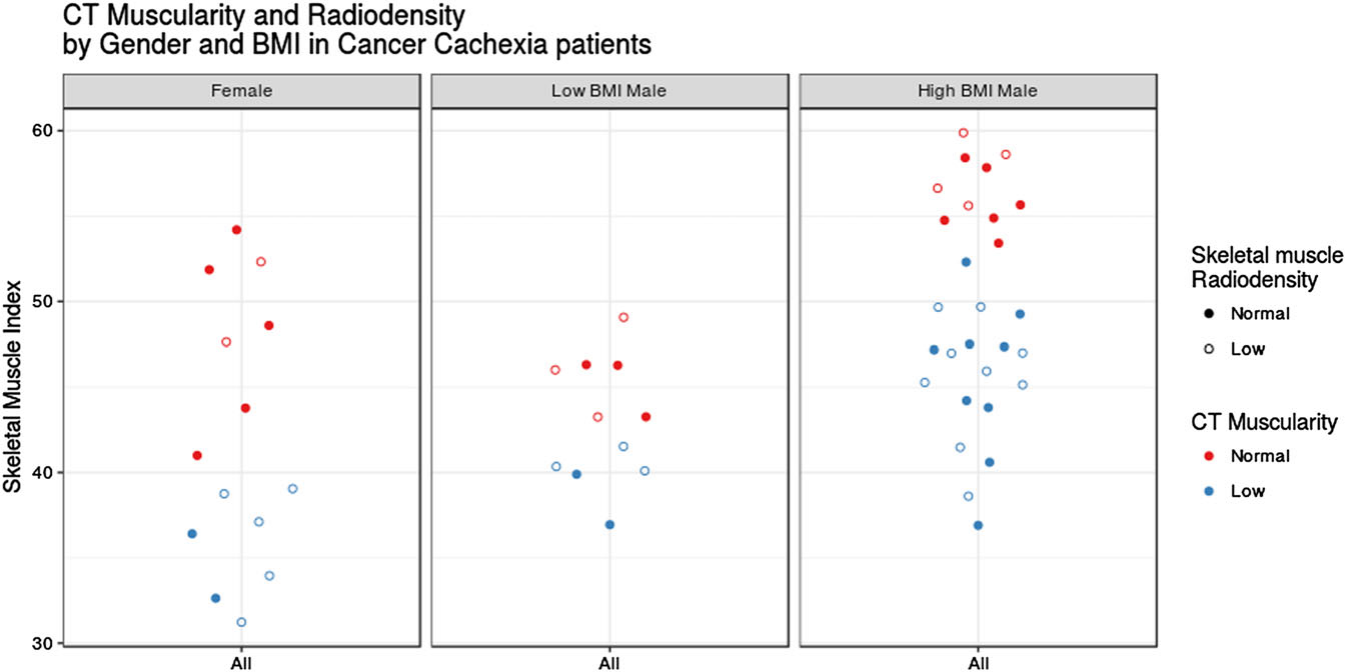

**2–67**

**Patient‐reported quality of life has no clear relationship with CT‐derived body composition**

Michael I. Ramage*, Edi Schuepbach, Ronnen Roubenof, D.A. Christopher Deans and Stephen J. Wigmore and James A. Ross and Carsten Jacobi and Richard J.E. Skipworth

*Department of Clinical Surgery, Royal Infirmary of Edinburgh, Scotland, UK*

**Introduction:** Body composition analysis by CT has been proposed as an outcome measure in trials of anti‐cachexia interventions. However, the relationship between CT‐variables and patient quality of life (QoL) is unexplored.

**Methods:** CT planimetry at L3 was performed on preoperative scans of patients with upper GI cancer (UGIC: *n* = 89; M:F 62:27; median 68 yrs), vascular/aortic surgery (AAA: *n* = 52; M:F 44:8; median 68 yrs), and healthy live kidney donors (LDN: *n* = 53; M:F 24:29; median 52 yrs). CT muscularity was measured using skeletal muscle index (SMI) with following cut points to define low muscularity (LM): <41cm^2^/m^2^ in females; <43cm^2^/m^2^ in BMI < 25 males; <53cm^2^/m^2^ in BMI > 25 males (Martin et al. 2013). SMD was also assessed in UGIC patients. Quality of life (QoL) was assessed by QLQ‐C30 symptomatic and functional domains, with clinical significance indicated by a 10 point difference in score.

**Results:** CT‐derived LM was prevalent in UGIC 47%; LDN 40%; AAA 56%, and low SMD was present in 53% UGIC. Cachexia was present in 39/62 (63%) UGIC males and 14/27 (52%) UGIC females. Contrary to anticipated observations, relative improvements in QoL were noted in UGIC patients with low SMI. In particular, physical functioning was improved with low SMI in UGIC females (96.67 v 83.33, *p* = 0.0036), and financial difficulties were also reduced (0 v 20.83, *p* = 0.036), whereas trouble with coughing was improved in low SMI in UGI males (19.54 v 32.10, *p* = 0.04). However, dyspnoea symptoms were worse in male patients with low SMD (13.33 v 2.47, *p* = 0.019). In non‐cancer populations, emotional functioning was improved with low SMI in male AAA patients (90.67 v 75, *p* = 0.027), whereas no clinically significant relationships were observed between QoL and SMI in healthy LDN patients.

**Discussion:** CT‐derived low muscularity has no explicable relationship with global QoL. Further studies are required to validate LM as a patient‐centred outcome measure.

**2–68**

**The emerging disconnect between muscle mass and function: evidence from different patient populations**

Michael I. Ramage*, Janice Miller, Edi Schuepbach, Ronnen Roubenof and D.A. Christopher Deans and Stephen J. Wigmore and James A. Ross and Carsten Jacobi and Richard J.E. Skipworth

*Department of Clinical Surgery, Royal Infirmary of Edinburgh, Scotland, UK*

**Introduction:** Recent clinical trials of anti‐cachexia interventions (including the ROMANA [anamorelin] and POWER [enobosarm] trials) have been unsuccessful, as although the interventions have resulted in increased lean body mass, there were no significant improvements in the functional co‐primary endpoints. We aimed to investigate the relationship between CT‐derived muscularity and muscle function in cancer and control patients.

**Methods:** Cachexia was defined using the consensus definition (Fearon et al. 2011). CT planimetry at L3 was performed on preoperative scans of upper GI cancer (UGIC), vascular/aortic surgery (AAA), and live kidney donor (LDN) patients. CT muscularity was measured using skeletal muscle index (SMI) with following cut points to define low muscularity (LM): <41cm^2^/m^2^ in females; <43cm^2^/m^2^ in BMI < 25 males; <53cm^2^/m^2^ in BMI > 25 males (Martin et al. 2013). SMD was also assessed in UGIC patients. Objective physical function was assessed by timed up‐and‐go (TUG).

**Results:** A total of 197 patients were recruited: UGIC *n* = 89; M:F 62:27; median 68 yrs; AAA *n* = 52; M:F 44:8; median 68 yrs; LDN *n* = 53; M:F 24:29; median 52 yrs. CT‐derived LM was prevalent in UGIC 47%; LDN 40%; AAA 56%. Low SMD was present in 53% of cancer patients. Cachexia was present in 39/62 (63%) males and 14/27 (52%) females. Median TUG was normal (<20s) in all patient groups (UGIC:8.68 s[range 5.18–18.65], LDN:7.72 s[5.73–11.22], AAA:9.5 s[5.99–14.66]).

**Discussion:** Despite the prevalence of LM and cachexia in the cancer patients, TUG times were still normal, suggesting that the patients were functionally unimpaired and non‐frail. This observation is entirely consistent with the pre‐operative medical assessment that these patients were fit for surgery, and was confirmed in the non‐cancer cohorts. These results confirm the hypothesis that cachexia can exist in the absence of functional impairment, and support the observation that an increase in LBM may not translate into functional improvement. We hypothesise that the relationship between muscularity and functional status is:

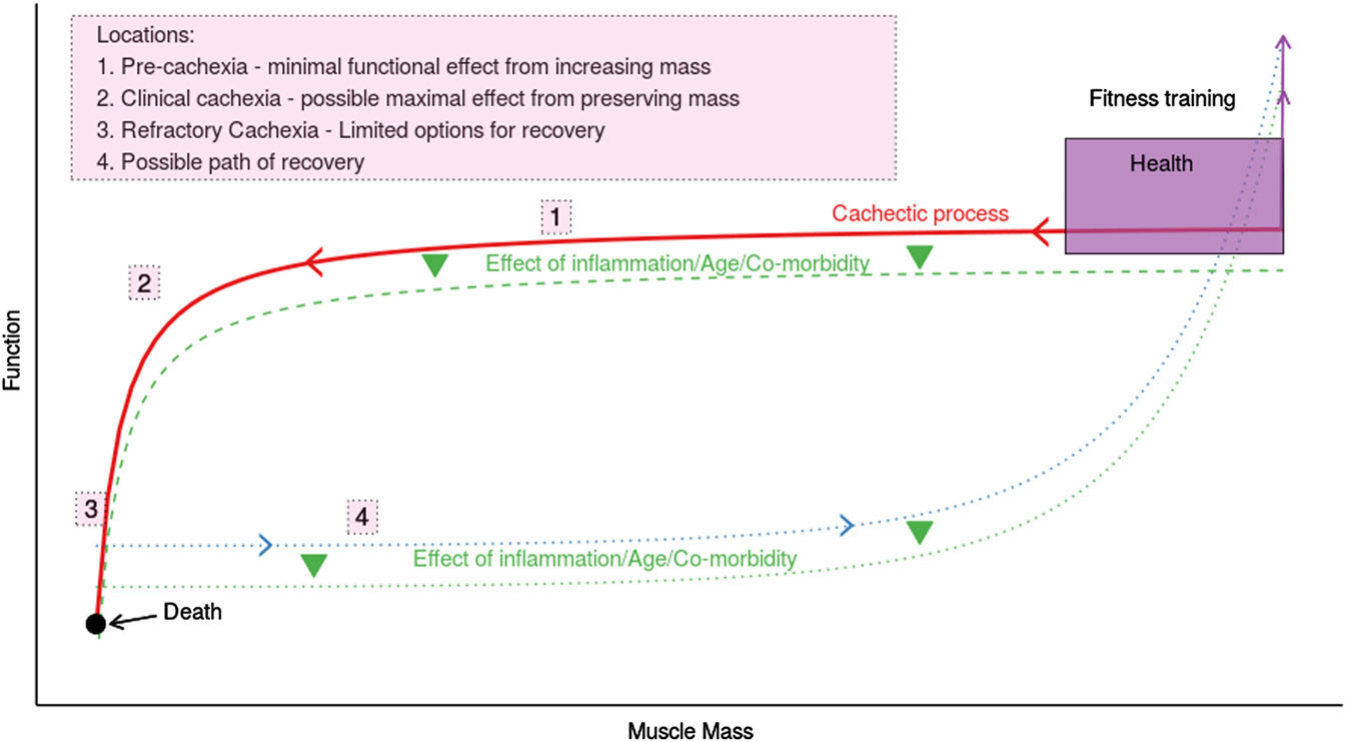

**2–69**

**Whole body calorimetry use in cancer patients: A case study of agreement between measured and predicted energy needs**

Ilana Roitman*^1,2^, Sarah A. Purcell^1^, Claire F. Trottier^1^ and Carla M. Prado^1^

^1^ *Human Nutrition Research Unit, Department of Agricultural, Food, and Nutritional Science, University of Alberta;* ^2^ *Cancer Institute of Sao Paulo, Faculty of Medicine Foundation of the University of Sao Paulo, Brazil*

**Introduction:** Inaccurate estimation of energy needs places cancer patients at risk for weight loss, malnutrition and cachexia. Muscle mass is likely a major driver of changes in energy requirements during cancer. Here, we compared measured resting energy expenditure (REE) and total energy expenditure (TEE) assessed using a whole body calorimetry unit (WBCU) to values derived using predictive equations in two individuals with colorectal cancer, one with, one without sarcopenia.

**Methods:** Patients were recruited at the Cross Cancer Institute (AB, Canada), as part of an ongoing randomized controlled trial. Body composition was assessed by dual energy X‐ray absorptiometry (iDXA). Sarcopenia was defined by previously established cutpoints of low appendicular muscle mass. Patients stayed in the WBCU for 24 hours, where REE and TEE were measured. Four equations were used to predict REE. A stress factor (SF) of 1.3 and a thermic effect of food (TEF) of 1.1 were considered for the estimation of TEE. Energy intake guidelines for individuals with cancer (25–30 kcal/kg body weight/day) were compared with TEE.

**Results:** Patient 1 had a normal body weight and was sarcopenic; patient 2 had obesity but not sarcopenia. Most equations underestimated REE for both patients. Two commonly used equations would classify patient 2 as hypometabolic. By calculating TEE values using SF and TEF, patient 1 was more likely to have lower than predicted TEE compared to patient 2. Current dietary guidelines underestimated TEE for patient 1 and overestimated for patient 2.

**Conclusions:** This case study shows the accuracy of REE and TEE predictive equations is variable for individuals with cancer and affected by muscle mass. The inclusion of body composition into prediction equations may allow for more accurate estimation of energy requirements in clinical settings, benefiting cancer patients at need for individualized nutrition intervention.

**2–70**

***IL‐*10 haplotypes and TNF‐α levels are associated with low muscle mass in patients with chronic hepatitis C**

Tatiana Bering^1^, Kiara Gonçalves Dias Diniz^1^, Marta Paula Pereira Coelho^1^, Diego Alves Vieira^1^, Adriana Dias Gomes^2^, Adriana Maria Kakehasi^3^, Rosângela Teixeira^1,4^, Dulciene Maria Magalhães Queiroz^2^, Gifone Aguiar Rocha^2^ and Luciana Diniz Silva*^1,4^

^1^ *Outpatient Clinic of Viral Hepatitis, Instituto Alfa de Gastroenterologia, Faculdade de Medicina, Universidade Federal de Minas Gerais, Belo Horizonte, Minas Gerais, Brazil;* ^2^ *Laboratory of Research in Bacteriology, Faculdade de Medicina, Universidade Federal de Minas Gerais, Belo Horizonte, Minas Gerais, Brazil;* ^3^ *Locomotor System Department, Faculdade de Medicina, Universidade Federal de Minas Gerais, Belo Horizonte, Minas Gerais, Brazil;* ^4^ *Department of Internal Medicine, Faculdade de Medicina, Universidade Federal de Minas Gerais, Belo Horizonte, Minas Gerais, Brazil*

**Introduction**: Despite the negative impact of low muscle mass (MM) on survival of cirrhotic patients, the mechanisms linked to MM loss are not completely understood in patients with chronic hepatitis C (CHC). Therefore, our aim was to evaluate whether the *IL‐10* haplotype (−1082G > A, ‐819C > T, and ‐592C > A) and serum levels of tumour necrosis factor‐alpha (TNF‐α) were associated with low MM in CHC patients.

**Methods:** 94 consecutive CHC outpatients (mean age, 50.3 ± 11.5 yrs.; 74.5% males; 68.1% without cirrhosis and 31.9% with compensated cirrhosis) and 164 healthy controls were prospectively enrolled. SNPs were genotyped by RT‐PCR. Serum levels of TNF‐α were measured by ELISA. CHC patients, prospectively, underwent scanning of the lean tissue, appendicular skeletal muscle mass (ASM) and fat mass by dual‐energy X‐ray absorptiometry. The data analysed included appendicular skeletal mass (ASM) standardized for height (ASMI = ASM/height^2^). The cut‐off points for low ASMI were 5.45 kg/m^2^ and 7.26 kg/m^2^ for women and men, respectively, according to Baumgartner *et al*. (1998). The International Physical Activity Questionnaire was used to determine the physical activity level.

**Results:** *IL10* SNPs were in Hardy Weinberg equilibrium. Patients and healthy subjects showed the same distribution of genotypes. Low ASMI was found in 12/94(12.8%) of the patients with CHC. The *IL‐10* haplotype ATA (low‐producer genotype) was observed in 11/12 (91.7%) of the patients with low ASMI (*P* = 0.03) and in only one of the patients without low ASMI 1/12 (8.3%). In the multivariate analysis, low ASMI was significantly and independently associated with moderate‐to‐high physical activity (OR = 0.31; 95%CI = 0.09–0.98; *P* = 0.05), TNF‐α levels (OR = 1.06; 95%CI = 1.01–1.11; *P* = 0.02) and ATA haplotype (OR = 9.87; 95%CI = 1.13–94.85; *P* = 0.05).

**Conclusions:** This is the first study to demonstrate that the *IL10* haplotype is associated with low ASMI in CHC patients. We also demonstrated that TNF‐α is associated with low ASMI in CHC patients.

**2–73**

**MRI‐based body composition profiling of sarcopenia shows association with prior health care burden in a large‐scale population study**

Janne West^1^, Jennifer Linge^1^, Jimmy D. Bell^2^, E. Louise Thomas^2^, Magnus Borga^1^ and Olof Dahlqvist Leinhard*^1^

^1^ *Advanced MR Analytics AB, Linköping, Sweden;* ^2^ *Research Centre for Optimal Health, Department of Life Sciences, Faculty of Science and Technology, University of Westminster, London, UK*

**Introduction:** Sarcopenia is often defined using appendicular lean tissue normalized with height^2^, measured by DXA. An alternative approach may be the use of MRI‐based thresholds, particularly as a combination with emerging body composition profiling techniques. In the current study, MRI‐based thresholds were used to define sarcopenia in a large‐scale population study, and associations of body composition measurements with prior health care burden were calculated.

**Methods:** 5.681 subjects (3.086 females, 2.595 males) were included from the imaging sub‐cohort of UK Biobank. All subjects were scanned using a neck‐to‐knee Dixon MRI protocol on a 1.5 T MR‐scanner. Body composition profiling was performed with AMRA® Profiler (AMRA‚ Sweden) measuring visceral adipose tissue index (VATi = VAT/height^2^), abdominal subcutaneous adipose tissue index (ASATi = ASAT/height^2^), total thigh muscle volume index (TTVi = TTV/height^2^), liver proton density fat fraction (PDFF), and intra‐muscular fat in the anterior thigh muscles (IMAT). Gender‐specific TTVi thresholds for sarcopenia, based on comparisons with DXA, were used (TTVi <3.64 l/m^2^ for men and TTVi <2.76 l/m^2^ for women) maximizing sensitivity and specificity. Finally, associations between the MRI‐acquired body composition measurements and prior health care burden were calculated, controlling for sex and age. Health care burden was defined as number of night's hospitalization up to ten years prior to the MRI‐scan, truncated at 30 nights, excluding gestational‐related hospitalization.

**Results:** 986 subjects were defined as Sarcopenic using MRI‐based TTVi thresholds, sensitivity and specificity compared to DXA was 0.88. In this group VATi and IMAT were associated with prior health care burden (*p* = 0.011, OR = 0.34 and *p* < 0.001, OR = 1.99 respectively). No significant associations were found for ASATi, TTVi or liver fat fraction.

**Conclusions:** MRI‐based defined sarcopenia allow the inclusion in rapid body composition MR‐scans enabling a detailed view of the patient's disease state. Body composition profiling revealed a strong association between IMAT and prior health care burden for the sarcopenic group.

**2–74**

**Sarcopenic obesity and functional capacity and clinical outcome in patients with heart failure using different diagnostic criteria: results from the Studies Investigating Co‐morbidities Aggravating Heart Failure (SICA‐HF)**

Masakazu Saitoh*^1^, Nicole Ebner^1^, Jochen Springer^1^, Stefan D. Anker^1,2^ and Stephan von Haehling^1^

^1^ *Department Cardiology and Pneumology, University Medical Center Göttingen, and DZHK (German Center for Cardiovascular Research), Göttingen, Germany;* ^2^ *Division of Cardiology and Metabolism – Heart Failure, Cachexia & Sarcopenia, Department of Cardiology (CVK); and Berlin‐Brandenburg Center for Regenerative Therapies (BCRT); Deutsches Zentrum für Herz‐Kreislauf‐Forschung (DZHK) Berlin, Charité Universitätsmedizin Berlin, Germany*

**Background:** We aimed to assess the association between sarcopenic obesity and functional capacity, clinical outcome in patients with heart failure (HF) using different diagnostic criteria for obesity.

**Methods:** We studied 251 patients with HF (51 females, 67 ± 11 years) were recruited as a part of the Studies Investigating Co‐morbidities Aggravating Heart Failure (SICA‐HF) program. Sarcopenia was defined as either a gait speed of <0.8 m/s or reduced handgrip strength (<30 kg in males and <20 kg in females), together with loss of skeletal muscle mass, i.e., appendicular skeletal muscle mass two standard deviations below the mean of a healthy reference group aged 18–40 years. Obesity was defined by three different diagnostic criteria, 1) body mass index (BMI > 30 kg/m^2^), 2) waist circumference (WC; >90 cm in males and >85 cm in females), and 3) body fat percentage (% fat; >30% in males and >40% in females). Sarcopenic obesity was considers as he combination of sarcopenia and obesity according to each definition. Functional capacity was assessed as peak oxygen uptake (peak VO_2_), 6‐minute walk test, and short physical performance battery (SPPB).

**Results:** Prevalence of sarcopenic obesity was 2.8% (BMI criteria), 6.8% (% fat criteria), and 11.7% (WC criteria). Sarcopenic obesity defined by WC and %fat criteria had significantly lower value of peak VO_2_, 6‐minute walk distance, and SPPB score than normal and obese alone group (all *p* < 0.05). There were 28 deaths during a 24‐month follow‐up. Sarcopenic obesity by any diagnostic criteria was not statistically significantly associated with all‐cause mortality during follow‐up.

**Conclusions:** Sarcopenic obesity defined by WC and body fat diagnostic criteria for obesity was associated with impaired functional capacity in patients with HF, however sarcopenic obesity defined by any diagnostic criteria were not sufficient to increase mortality.

**3–18**

**Muscle‐derived exosome/miRNA‐26a attenuates skeletal muscle wasting and cardiomyopathy in chronic kidney disease mice**

Bin Wang^1,2^, Aiqing Zhang^1,3^, Janet Klein^1^ and Xiaonan H. Wang*^1^

^1^ *Renal Division, Department of Medicine, Emory University, Atlanta, GA, USA;* ^2^ *Institute of Nephrology, Zhong Da Hospital, Southeast University, Nanjing, China;* ^3^ *Department of Paediatric Nephrology, Nanjing Medical University, Nanjing, China*

**Introduction:** Uremic cardiomyopathy and muscle atrophy contribute to CKD‐induced morbidity and mortality. Exosomes, natural carriers of many signal molecules including microRNA (miR), mediate organ to organ communication. We hypothesized that miR‐26 would benefit both CKD‐induced muscle wasting and cardiomyopathy through exosome‐mediated muscle‐heart crosstalk.

**Methods:** We used an engineered exosome vector which contains an exosomal membrane protein gene Lamp2b fused with muscle specific surface peptide for targeting delivery. Exosome encapsulated miR‐26a precursor RNA (Exo/miR26) were generated in muscle satellite cells and injected into the tibialis anterior (TA) muscle of CKD mice (5/6 subtotal nephrectomy) for 12 weeks. NanoSight instrument was used to quantify exosomes. A miR deep sequencing assay and qPCR were used to identify microRNA expression. Cardiac ultrasound was used to detect heart size and function. In‐Vivo Xtreme camera system was used to detect exosome in vivo.

**Results:** miR‐26a was decreased in skeletal muscle and heart of CKD mice. Uremic serum enhanced secretion of miR‐26a exosomes in cultured C2C12 skeletal and H9C2 cardiac muscle cells. The intervention of Exo/miR26a increased the expression of miR‐26a in skeletal muscle and heart, as well as increased muscle cross‐section area and decreased CKD‐induced upregulation of atrogin‐1 and MuRF1. Curiously, cardiac fibrosis lesion was partially depressed, and FoxO1, α‐SMA, connect tissue growth factor (CTGF), fibronectin and collagen1α were decreased in CKD mice with intramuscular injection of Exo/miR‐26a. Echocardiography showed that the percentage of ejection fraction was increased in CKD mice treated with Exo/miR26a. Using fluorescence dye labeled Exo/miR26a, we found that the fluorescence intensity in heart was correlated with skeletal muscle, examined by linear regression. We found that miR‐26a directly inhibits FoxO1 and CTGF, which provided mechanism for inhibition of muscle atrophy and cardiac fibrosis by Exo/miR26a.

**Conclusions:** overexpression of miR‐26a in muscle prevents CKD‐induced muscle loss and attenuates cardiac fibrosis via exosome‐mediated muscle‐heart crosstalk.

**Funding:** NIH R01 AR060268.

**3–34**

**Blunted activation of satellite cells in parallel with up‐regulated proliferation of connective tissue fibroblasts in ageing human skeletal muscle undergoing acute regrowth and regeneration**

Ulrik Frandsen*^1^, Rikke S. Kamper^1^, Per Aagaard^1^, Anders N. Jørgensen^1^, Lars G. Hvid^1^, Michael Kjaer^2^ and Charlotte Suetta^3^

^1^ *Institute of Sports Science and Clinical Biomechanics, University of Southern Denmark;* ^2^ *Institute of Sports Medicine and Center of Healthy Aging, Faculty of Health, University of Copenhagen, Bispebjerg Hospital, Denmark;* ^3^ *Dept of Clinical Physiology and Nuclear Medicine & PET, Rigshospitalet, University of Copenhagen Denmark & Division of Clinical Physiology and Nuclear Medicine, Department of Diagnostics, Glostrup University Hospital, Denmark*

**Background/aims**: The cellular mechanisms responsible for the attenuated regenerative capacity in ageing human skeletal muscle are not fully understood. Here we examined the acute activation of muscle satellite cells (SC) and connective tissue fibroblast (Tcf4) in young and older human subjects after 4 days of immobility‐induced muscle atrophy and again after 6 days of re‐ambulation.

**Methods:** Myofiber atrophy was induced by knee‐bracing for a period of 4 days in young (YM, ~20 yrs., *n* = 9) and aged (OM, ~70 yrs., n = 9) male adults. Muscle regrowth after atrophy was induced by 6 days of re‐ambulation supplemented by 2 sessions of supervised unilateral resistance training for the disused leg. Measures of SC and Tcf4 expression were analysed in muscle biopsies (VL) obtained Pre, after 4 days immobility (4d–Imob) and after additional 6 days of re‐mobilization (6d–REmob). SCs were quantified and expressed as a percentage of total myonuclei (SC/MN) in MHC‐I and MHC‐II sensitive immunoflourescent stainings of muscle cross sections to determine fiber type, with Pax‐7 as a marker of SCs, and DAPI and laminin stainings performed to verify the spatial location of SC and myonuclei.

**Results:** We report that SC in type I fibers increased in YM and OM at 6d–REmob compared to Pre and 4d–Imob (*P* < 0.01). Further, SC in type II fibers increased in YM with no change in OM at 6d–REmob compared to Pre and 4d–Imob (*P* < 0.01). An overall effect of age was found for SC cell content in relation to type I and type II fibers (*P* < 0.01), thus demonstrating blunted SC proliferation in OM, indicating an impaired regenerative capacity. To further describe the SC pool, we determined the proportion of active SCs using NCAM, Ki67 and DAPI staining, with activated SCs identified as both NCAM and Ki67 positive (NCAM^+^/Ki67^+^). NCAM^+^/Ki67^+^ SC's increased at 6d–REmob in YM but not OM (4d–Imob:YM 0.6 ± 0.5/100 fibers; OM 0.2 ± 0.4/100 fibers & 6d–Rmob: YM 2.5 ± 1.2/100 fibers; OM 0.6 ± 0.5/100 fibers. *P* < 0.05). However, a markedly elevated expression of NCAM^−^/Ki67^+^ cells was noted in OM after remobilization. Further analysis of Tcf4 expression revealed that connective tissue fibroblast (Tcf4^+^/Ki67^+^) became markedly more activated in OM compared to YM at 6d–REmob compared to 4d–Imob (4d–Imob: OM 1.1 ± 1.3 /100 fibers; YM 2.6 ± 1.5/100 fibers & 6d–Rmob: OM 7.2 ± 7.5/100 fibers; YM 2.8 ± 1.7/100 fibers. *P* < 0.05).

**Conclusions:** The present study demonstrates a blunted activation of skeletal muscle satellite cells in parallel with up‐regulated proliferation of Tcf4 connective tissue fibroblasts in ageing human skeletal muscle with acute regeneration. Our data support the proposition that progressive fibrosis with ageing may inhibit proliferation of myogenic progenitor cells (SC's) and thereby contribute to the progressive impairment in regenerative capacity of human skeletal muscle with aging and disease.

**3–35**

**Molecular characterization of novel serous ovarian cancer variant associated with myosteatosis**

Cynthia Stretch*^1^, Vera C. Mazurak^2^, Gregg Nelson^1,3^ and Oliver F. Bathe^1,4^

^1^ *Department of Oncology, University of Calgary, Calgary, Alberta, Canada;* ^2^ *Department of Agricultural Food and Nutritional Science;* ^3^ *Department of Obstetrics & Gynecology, University of Calgary, Calgary, Alberta, Canada;* ^4^ *Department of Surgery, University of Calgary, Calgary, Alberta, Canada*

**Introduction**: Ovarian cancer is the fifth most common cause of cancer death in women. Decreased muscle radiodensity is a consequence of ovarian cancer associated with truncated disease‐free and overall survival. We postulated that the molecular features of the tumour drive changes in muscle radiodensity/myosteatosis.

**Methods**: Microarray and RNASeq data from serous ovarian cancer patients (*n* = 514) was obtained from The Cancer Genome Atlas Project. Body composition analysis was conducted for patients with available computed tomography scans at L3 (*n* = 112). Myosteatosis was assigned if patients had an L3 mean muscle radiodensity <30 HU. A predictive model was built to classify patients without a scan as myosteatosis‐associated variant positive (MAV+) or negative (MAV‐), using orthogonal partial least squares discriminant analysis. We then studied transcriptional differences between MAV+ and MAV‐.

**Results**: MAV+ patients had significantly decreased disease free and overall survival (Log rank p‐value <0.05) and did not significantly differ in muscle or adipose surface area at L3. A model distinguishing MAV+ and MAV‐ patients (R2 = 0.954; Q2 = 0.802; CV‐ANOVA *p* = 1.17E‐32) was validated using two independent external validation datasets. Differential expression showed tumors from MAV+ patients had increased cell cycle progression, increased proliferation, increased survival, decreased apoptosis, increased insulin signaling, decreased triacylglycerol degradation, increased lipid biosynthesis.

**Conclusions:** We have identified a novel variant of serous ovarian carcinoma associated with a bad prognosis and with gross effects on the health of the host (myosteatosis). We provide a picture of the molecular features of ovarian cancers that are associated with this novel variant. Our data suggests tumors from MAV+ patients are more proliferative, resistant to cell death and have altered energy metabolism compared to tumors from MAV‐ patients. If this variant preferentially uses lipids as fuel, then inhibition of related metabolic pathways would not only inhibit tumour growth, but also simultaneously enhance the health of the host.

**3–36**

**Six1, Eya1, Esrrb and Pax3 are stably expandable induced myogenic stem cells**

Eun‐Joo Lee and Kyu‐Shik Jeong*

*Department of Pathology, College of Veterinary Medicine, Kyungpook National University, Daegu City, Republic of Korea*

**Introduction:** Life‐long regeneration of healthy muscle by cell transplantation is an ideal therapy for patients with degenerative muscle diseases. Yet, obtaining muscle stem cells from patients is very limited due to their exhaustion in disease condition. Thus, development of a method to obtain healthy myogenic stem cells is required.

**Methods:** Here, we showed that the four transcription factors, Six1, Eya1, Esrrb and Pax3, converts fibroblasts into induced myogenic stem cells (iMSCs).

**Results:** The iMSCs showed effective differentiation into multinucleated myotubes and also higher proliferation capacity than muscle derived stem cells both in vitro and in vivo. The iMSCs do not lost their proliferation capacity though the passaging number is increased. We further isolated CD106‐negative and α7‐integrin‐positive iMSCs (sort‐iMSCs) showing higher potent myogenic differentiation capacity than iMSCs. Moreover, genome‐wide transcriptomic analysis of iMSCs and sort‐iMSCs, followed by network analysis, revealed the genes and signaling pathways associated with enhanced proliferation and differentiation capacity of iMSCs and sort‐iMSCs, respectively.

**Conclusions:** The stably expandable iMSCs provide new source for drug screening and muscle regenerative therapy for muslce wasting disease including sarcopenia.

**3–37**

**Relationship between muscle mass and usual walking speed mediated by muscle strength, depression, and respiration in younger elderly women: preliminary study**

Yun‐jeong Baek*^1^ and Chung‐hwi Yi^2,3^

^1^ *Dept. of Physical Therapy, College of Health Science, Yonsei University;* ^2^ *Dept. of Physical Therapy, College of Health Science, Yonsei University;* ^3^ *Dept. of Ergonomic Therapy, The Graduate School of Health and Environment, Yonsei University*

**Introduction**: The phenomenon concerning the gradual, age‐related decrease of skeletal muscle mass and strength is called *sarcopenia*. The European Working Group on Sarcopenia in Older People (EWGSOP) suggests that sarcopenia has three stages. During pre‐sarcopenia to severe sarcopenia, a reduction in muscle mass occurs, as well as a decrease in strength and physical performance. Among the strength measurement factors suggested by EWGSOP, knee extension strength (KES), hand grip strength (HGS), and peak expiratory flow (PEF) are correlated with muscle mass and usual walking speed (UWS). In addition, the psychological factor of depression correlates with muscle mass and UWS. However, few studies have reported a correlation between muscle mass and UWS. Therefore, we hypothesized that the effect of muscle mass on UWS may be mediated by KES, HGS, PEF, and depression.

**Method**: In this study, median analyses were used to assess the median effect. Median analyses were progressed in SPSS ver.23 using a downloaded INDIRECT macro. A total of 100 women aged 65 to 80 years were surveyed.

**Results**: Because of a 5,000 bootstrap sampling, KES [bias corrected (BC) 95% confidence interval (CI): 0.0033~0.0246] and PEF (BC 95% CI: 0.0020~0.0217) did not contain zero.

**Conclusions:** It was found that KES and PEF are the significant mediator between the muscle mass and UWS.

**3–38**

**Gender differences in general health condition of older adults: the VERISAÚDE study**

José C. Millán‐Calenti*^1^, Rocio López‐López^1^, Laura Lorenzo‐López^1^, Carmen de Labra^1^, David Facal^2^ and Ana Maseda^1^

^1^ *Gerontology Research Group. Institute of Biomedical Research (INIBIC), University of A Coruña, A Coruña, Spain;* ^2^ *Applied Cognitive Neuroscience and Psychogeriatric Group (NeuCogA‐Aging), University of Santiago de Compostela, Santiago de Compostela, Spain*

**Introduction:** Gender‐related differences in socio‐demographic, medical, psychological and functional conditions were evaluated in older adults living in the community.

**Methods**: This multicenter study was conducted as part of the VERISAÚDE project, a prospective cross‐sectional population‐based study of community‐dwelling individuals aged 65 years and over enrolled in senior community centers of North West Spain (*n* = 749). From 2013 to 2014, a comprehensive gerontological assessment (CGA) was used to assess the social, medical, psychological and functional needs and risk factors of this representative population.

**Results**: Significant gender‐related differences were not found in visual impairment, self‐rated health and instrumental activities of daily living. Males showed significantly higher prevalence than females of hearing impairment, toxic habits, comorbidity, and better social resources and quality of life, while females showed a higher prevalence of frailty, higher risk of malnutrition, more drug consumption and polypharmacy, poor cognitive scores, and higher depressive scores. Significant gender‐related differences were not found in visual impairment, self‐rated health and instrumental activities of daily living.

**Conclusions**: Gender differences were observed in different health variables. We conclude that CGA is a valid multidimensional diagnostic instrument to identify, quantify and manage the needs of older adults living in the community. Importantly, gender‐related differences should be specifically explored and taking into account when developing socio‐sanitary measures to promote active aging and improve the quality of life of older adults. In future longitudinal studies, it should be explored whether these differences are the result of the different level of contact with risk factors, or if they result from a different gender‐related reaction to the same risk factors.

*This work was supported by the Xunta de Galicia, FrailNet network IN607C 2016/08*.

**3–39**

**Age‐and gender‐related differences in myosin heavy chain isoforms with muscle strength, quality and function**

Seung‐Lyul Oh*^1,2^ and Jae‐Young Lim^1,2^

^1^ *Dept. of Rehabilitation Medicine, Aging & Mobility Biophysics Lab, Seoul National University Bundang Hospital, Korea;* ^2^ *Institute on Aging in Seoul National University, Korea*

**Introduction:** Declining muscle strength and function are hallmarks of the aging process. In the present study, we determined the gender related to differences in Myosin Heavy Chain (MHC) isoforms, muscle strength and function with aging.

**Methods:** The study recruited total 53 healthy participants (32 male, 21 female) aged 20–85 years, who were divided into four group; young men (*n* = 17, YM, 29.23 ± 4.51), older men (*n* = 15, OM, 71.87 ± 3.42), young women (*n* = 11, YW, 29.64 ± 4.88), older women (*n* = 10, OW, 68.1 ± 1.91). We analysed body composition (by DEXA), lower body muscle strength and quality, and function (by isokinetic device). We took the vastus lateralis from all participants for analyzing the type of MHC isoforms.

**Results:** The men showed a greater age‐related decline in skeletal muscle mass (18.6%, *p* < 0.01), lean body mass (10.1%, *p* < 0.05), grip strength (35.3%, *p* < 0.001), isometric strength (29.6%, p < 0.001), isotonic power (42.5%, p < 0.001), isokinetic strength (44.3%, p < 0.001), and muscle quality (24.8%, *p* < 0.01), whereas the women showed a significant lower only in isometric strength (24.2%, *p* < 0.05) and isotonic power (28.3%, *p* < 0.01) with aging. In addition, the proportion of MHC IIa was significantly lower in both OM (p < 0.05) and OW (p < 0.05) than in YM and YW, respectively. However, only OM showed a significant larger in MHC‐I (p < 0.01) than YM, and high proportion of MHC‐I found in both younger and older women. Also, there was negative correlation between MHC‐I isoform and isokinetic strength and muscle quality.

**Conclusions:** These results show that gender related to differences seem to exist in muscle mass, strength, and function with advancing the aging. The influence of muscle strength and function on aging significant affects in men, not women. Larger proportion of MHC‐I are associated with lower muscle strength and function, especially in women with younger age.

**3–40**

**Sexual dimorphism in the skeletal muscle transcriptome and urinary proteome indicate sex specific pathways involved in regulation of muscularity in cancer patients**

Cynthia Stretch*^1^, Karen Wang^2^, Tomas Rejtar^2^, Stefan Reinker^2^, Sophie Brachat^2^, Ralf Badur^2^, David Glass^2^, Didier Laurent^2^, Sambasivarao Damaraju^3,4^, Oliver F. Bathe^1,5^, Ronenn Roubenoff^2^, Carsten Jacobi^2^ and Vickie E. Baracos^3^

^1^ *Department of Oncology, University of Calgary, Canada;* ^2^ *Novartis Institutes for BioMedical Research Basel, Novartis Pharma AG, CH‐4056 Basel, Switzerland and Novartis Institutes for Biomedical Research, Cambridge, Massachusetts, USA;* ^3^ *Department of Oncology, University of Alberta, Canada;* ^4^ *Department of Laboratory Medicine and Pathology, University of Alberta, Canada;* ^5^ *Department of Surgery, University of Calgary, Canada*

**Introduction**: Sexual dimorphism exists in skeletal muscle mass, fiber type, fiber size and response to diseases such as cancer. Our hypothesis is that variations in muscularity in men and women with cancer are associated with different molecular signatures.

**Methods**: Molecular data were obtained from 41 K Agilent microarray analysis of *rectus abdominus* muscle samples (*n* = 94, 55♂, 39♀) and LC–MS analysis of urine samples (*n* = 112, 63♂, 49♀) from patients with cancer. Computed tomography images were used to measure the skeletal muscle index (SMI) for all patients in the study. Female and male patients were classified as sarcopenic if their SMI <38.5 cm^2^/m^2^ and <52.4 cm^2^/m^2^, respectively. Differentially abundant features were identified and studied using pathway analysis software.

**Results**: Men were significantly more muscular (*p* < 0.0001) and had greater variation in muscularity than women. Table 1 shows a summary of results. We found little overlap in differentially abundant features in men and women. Muscularity was positively associated with proliferative pathways including JAK/STAT signaling in men and growth hormone signaling in women. Muscularity was negatively associated with mismatch DNA repair in men but not women, and with ER mediated phagocytosis in women but not in men. As with the muscle transcriptome, the urine proteome suggested altered carbohydrate metabolism in women, but not men. Finally, inflammation was associated with low muscle regardless of sex or data level. While we identified similar downstream features (e.g. NFkB signaling), there were differences in upstream signaling molecules. For example, sarcopenia was associated with increased IL25 expression and IL6 expression in men and women, respectively.

**Table 1:** Pathway analysis summary of differentially abundant features associated with muscularity.

	Muscle microarray†		Urine proteome^††^	
	Men	Women	Men	Women
Differentially abundant features, N	1356	835	27	64
Major pathways or molecules associated with muscularity*	‐ JAK/STAT signaling ↑ ‐ RNA translation ↑ ‐ actin cytoskeleton signaling ↑ ‐ RAC signaling ↑ ‐actin cytoskeleton signaling ↑ ‐FAK signaling ↑ ‐apoptosis ↓ ‐mismatch repair ↓ ‐ FOXO signaling ↓ ‐NFkB signaling ↓	‐ growth hormone signaling ↑ ‐ RNA translation ↑ ‐choline biosynthesis ↑ ‐gluconeogenesis ↑ ‐ ER mediated phagocytosis↓ ‐apoptosis ↓ ‐antigen presentation pathway ↓ ‐ FOXO signaling ↓ ‐ NFkB signaling ↓	‐ Structural cellular components ↑ ‐ Purine metabolism ↑ ‐ Lysosomal function components ↓ ‐ Cytokines ↓	‐ Motor subunit components ↑ ‐ Glycolytic enzymes ↑ ‐ Proteins involved in innate immune response ↑ ‐Purine metabolism ↑ ‐Coagulation cascade components ↓

† 72 genes were differentially abundant in both sexes; ^††^3 proteins were differentially abundant in both sexes.

* The arrow directions reflect the comparison ‘muscular versus sarcopenic’. For example, in men JAK/STAT signaling has a ‘↑’ because, based on differential expression, patients with more muscle had greater expression of JAK/STAT signaling genes.

**Conclusions:** Cancer associated muscle wasting involves decreased anabolism and increased catabolism. While our results agree with this, we find different anabolic and catabolic pathways are altered in sarcopenic muscle in men and women. Development of interventions for cancer‐associated wasting would benefit from a sex‐specific approach.

**3–41**

**Proportion of single frailty criteria in frail and pre‐frail older adults**

Rociao López‐López*^1^, José C. Millán‐Calenti^1^, Ana Buján^1^, José M. Cancela^2^, Ana Maseda^1^ and Laura Lorenzo‐López^1^

^1^ *Gerontology Research Group, Institute of Biomedical Research (INIBIC), University of A Coruña, A Coruña, Spain;* ^2^ *HealthyFit Research Group (HI22), University of Vigo, Pontevedra, Spain*

**Introduction**: Frailty geriatric syndrome is a dynamic process of increased vulnerability to stressors exposing the individual to a higher risk of negative health‐related outcomes. This status may represent a transition phase between successful aging and disability. In order to illustrate the extent to which pre‐frail older adults differed from frail older adults, the proportion of single frailty criteria met was compared between groups.

**Methods**: A cross‐sectional study was carried out covering a representative sample (*n* = 749) of older adults aged ≥65 years (mean age = 75.8 ± 7.2). Frailty status was diagnosed based on Fried's phenotypic definition (unintentional weight loss, exhaustion, low physical activity, low walking speed, and low grip strength). This instrument classifies people into pre‐frail (if they met 1–2 criteria), non‐frail (if no criteria were present), and frail (≥3 criteria) categories. Comparisons between pre‐frail and frail groups were performed by Chi‐square tests.

**Results**: As expected, results showed that the proportion of individual criteria was significantly higher in frailty compared with pre‐frailty (all *ps* < .001). “Low grip strength” was the most frequent criterion in the pre‐frail population (95.0%), followed by “slow‐walking time” (13.2%), “exhaustion” (6.7%), “unintentional weight loss” (4.6%), and “low physical activity” (1.7%). Low grip strength and slow walking time were also the two most frequent physical alterations in the frail group and the proportion of all criteria except low grip strength was strongly increased in frailty.

**Conclusions:** The fact that low grip strength was the criterion with more positive cases is relevant since weakness is considered as a warning sign of increasing vulnerability in early frailty development, and it has been shown to be a manifestation of the sarcopenia, possibly predicting dismobility syndrome. Thus, the prevention of sarcopenia is relevant for reducing the risk of frailty in elderly.

*This work was supported by the Xunta de Galicia, FrailNet network IN607C 2016/08*.

**3–43**

**Association between computed tomography assessment of skeletal muscle index and muscle attenuation and chemotherapy intolerance in patients with head and neck cancer: preliminary results**

Martine J. Sealy*^1,2^, Harriët Jager‐Wittenaar^1,2^, Tanadesh Dechaphunkul^3,7^, Wim P. Krijnen^1,4^, Cees P. van der Schans^1,5,6^, Jan L.N. Roodenburg^2^ and Vickie E. Baracos^3^

^1^ *Research Group Healthy Ageing, Allied Healthcare and Nursing, Hanze University of Applied Sciences, Groningen, The Netherlands;* ^2^ *Department of Oral and Maxillofacial Surgery, University of Groningen, University Medical Center, Groningen, The Netherlands;* ^3^ *Palliative Care Medicine, Department of Oncology, University of Alberta, Edmonton, AB, Canada;* ^4^ *Johan Bernoulli Institute for Mathematics and Computer Science, University of Groningen, Groningen, The Netherlands;* ^5^ *Department of Rehabilitation Medicine, University of Groningen, University Medical Center, Groningen, The Netherlands;* ^6^ *Department of Health Psychology Research, University of Groningen, University Medical Center, Groningen, The Netherlands;* ^7^ *Department of Otorhinolaryngology Head and Neck Surgery, Faculty of Medicine, Prince of Songkla University, Songkla, Thailand*

**Background:** It is unclear whether muscle mass depletion is associated with chemotherapy intolerance in head and neck cancer (HNC) patients. We aimed to assess the association between pre‐treatment computed tomography (CT) body composition measurements and chemotherapy intolerance in HNC patients.

**Methods:** Data were extracted from the oncological database of Northern Alberta, Canada, and analysed retrospectively. Adult HNC patients who had (surgery and) platin‐based chemo‐radiotherapy were included if a pre‐treatment CT scan at T4 or L3 level was available. Body composition was evaluated by assessment of skeletal muscle index (SMI; cm^2^/m^2^) and skeletal muscle radiation attenuation (MA; Hounsfield units), and was corrected for deviation from the mean, to enable merged T4 and L3 measurements. Chemotherapy intolerance was considered present if there was unplanned reduction or termination in chemotherapy regime ascribed to toxicity. Univariate and multivariate logistic regression analyses with SCAD best model selection were used to investigate associations between SMI or MA and chemotherapy intolerance. Multivariate analysis corrected for age, sex, smoking, drinking, body weight, BMI, comorbidity, location and stage of cancer, type of treatment and chemotherapy, and time between CT scan and start of chemo‐radiotherapy. A p‐level of <0.05 was considered significant and Odds Ratios (OR) [95% CI] were presented.

**Results:** 218 patients (age: 57.8 ± 10.3 y, male: 78%, T4 image: 45%, L3 image: 55%) were included. In the univariate analysis, no significant association with chemotherapy intolerance was found for SMI (*p* = 0.109, OR = 0.98 [0.96–1.00]) or MA (*p* = 0.547, OR = 0.99 [0.96–1.02]). Multivariate analysis identified a significant association between SMI and chemotherapy intolerance (*p* = 0.002, OR 0.95 [0.91–0.98]). The model included type of chemotherapy, BMI, interaction between smoking and drinking, and comorbidity.

**Conclusions:** Lower pre‐treatment values of SMI at T4 and L3 level are associated with higher occurrence of chemotherapy intolerance in HNC patients, suggesting that taking into account SMI may help preventing chemotherapy intolerance.

**3–44**

**The impact of aging and calorie restriction on mitochondrial morphology in oxidative and glycolytic skeletal muscles**

Julie Faitg*^1,3^, Jean‐Philippe Leduc‐Gaudet^2,3,4^, Olivier Reynaud^2,3^ and Gilles Gouspillou^2,3,5^

^1^ *Département de Biologie, Faculté des Sciences, UQAM, Québéc, Canada;* ^2^ *Département de Sciences de l'activité physique, Faculté des Sciences, UQAM, Québec, Canada;* ^3^ *Groupe de recherche en Activité Physique Adaptée, Québec, Canada;* ^4^ *Meakins‐Christie Laboratories, Department of Critical Care, McGill University;* ^5^ *Centre de Recherche de l'Institut Universitaire de Gériatrie de Montréal*

Skeletal muscle aging is associated with a progressive decline in muscle mass and strength, a biological process known as sarcopenia. A growing body of evidence indicates that mitochondrial dysfunction is causally involved in the development of sarcopenia. Calorie restriction (CR) is amongst the most efficient interventions to attenuate sarcopenia, and this strategy is thought to mediate its effects through its impact on mitochondria. However, many of the specific effects of CR on mitochondrial biology, especially in aged skeletal muscle, remain unclear or unknown. In particular, the effects of aging and CR on skeletal muscle mitochondrial morphology, which is known to greatly impact mitochondrial function, remain largely under investigated. The objectives of the present study were (i) to investigate the effects of aging on mitochondrial morphology in glycolytic and oxidative skeletal muscles and (ii) to define whether CR can attenuate the effects of aging on mitochondrial morphology. The following 3 groups of male Sprague Dawley rats were studied: 1‐ adult (9‐month‐old) ad‐libitum fed (AL); 2‐ old (21‐month‐old) AL fed and; 3‐ old (21‐month‐old) calorie restricted (CR; 40% reduction in food intake during 13 months) rats. The morphology of SubSarcolemmal (SS) and InterMyoFibrillar (IMF) mitochondria was assessed using a 2‐dimensional approach in the oxidative soleus (SOL) and glycolytic white gastrocnemius (WG) skeletal muscles. In the SOL, our results indicated that aging is associated with a fragmentation of both SS and IMF mitochondria. CR in the SOL seems to attenuate the aging‐related increase in mitochondrial fragmentation. In the WG, our results indicate that aged muscle displays enlarged SS mitochondria and more complex and branched IMF mitochondria. In the WG, CR did not attenuate the effects of aging. Our results therefore indicate that the effects of aging and calorie restriction on mitochondrial morphology are muscle specific.

**3–45**

**Role of dietary protein and exercise on tryptophan‐kynurenine metabolism in older patients during muscle disuse**

Barbara Strasser*^1^, Maria Hermanky^2^, Gabriele Kohlboeck^3^ and Dietmar Fuchs^4^

^1^ *Division of Medical Biochemistry, Biocenter, Medical University Innsbruck, Austria;* ^2^ *AUVA Lorenz Böhler Trauma Unit Vienna, Austria;* ^3^ *Austrian Society of Epidemiology, Innsbruck, Austria;* ^4^ *Division of Biological Chemistry, Biocenter, Medical University Innsbruck, Austria*

**Introduction:** Old age and frailty are associated with increased immune activation and alterations in tryptophan‐kynurenine metabolism. The activated immune system can be detected by increased neopterin and kynurenine to tryptophan concentrations. The aim of this prospective study was to evaluate the effect of a protein optimized diet on biomarkers of immune activation and tryptophan metabolism in older patients with hip fracture.

**Methods:** Forty participants (mean age: 79.5 yrs.) were randomly assigned to an intervention group (*n* = 20) or to a control group (n = 20). During hospitalization (mean length of stay: 16.4 d), the intervention group received a protein optimized diet (1.5 g/kg per day) and was instructed to moderate resistance training. The Mini Nutritional Assessment was used to assess malnutrition. Hand‐grip strength was measured using the JAMAR Dynamometer. Serum concentrations of tryptophan and kynurenine were determined by HPLC and immune system biomarker neopterin by ELISA at admission, discharge and at 1‐month follow‐up.

**Results:** At admission, 10.5% of the patients were malnourished and another 26.3% were at risk of malnutrition. The intervention group consumed more protein during hospitalization compared with the control group (*p* < 0.001): 70.5(SD 13.5)g/d *vs*. 52.3(SD 13.9)g/d. Older patients with hip fracture exhibit higher degrees of immune system activation as indicated by biomarker concentrations compared with reference values obtained from a healthy population (Table 1). None of the outcomes showed a significant intervention effect or interaction of intervention and time effect. Improvements in maximum hand‐grip strength after intervention were related to lower neopterin and higher tryptophan levels (*p* = 0.0113 and *p* < 0.0001, respectively).

**Conclusions:** Protein enrichment enabled older patients to increase protein intake to levels that are 80% of their optimized intake of 1.5 g/kg per day, however, did not affect immune‐systems biomarker and tryptophan metabolism during the early postoperative period. Muscularity may affect biochemical pathways which are linked to immune activation responses following injury.

**Table 1:** Changes in immune system biomarkers and tryptophan over time in 40 older patients with hip fracture compared with healthy controls.

**Total Group** **(*n* = 40)**	**Admission (A)**	**Discharge (D)**	**Follow‐up (F)**	**P‐values**			**Reference values** #
				**A vs. D**	**A vs. F**	**D vs. F**	
Tryptophan (μmol/L)	49.50 (13.74)	48.71 (13.79)	50.78 (14.80)	ns	ns	ns	67.4 (10.2)
Kynurenine (μmol/L)	2.41 (0.83)	2.78 (0.86)	2.73 (1.21)	0.010	0.032	ns	1.78 (0.42)
Kyn/Trp (μmol/mmol)	53.42 (26.44)	62.30 (27.08)	57.54 (32.98)	0.020	ns	ns	26.70 (6.2)
Neopterin (nmol/L)	11.95 (6.71)	17.73 (12.24)	14.36 (8.54)	<0.001	0.003	ns	5.94 (1.58)

Values are means ± standard deviation (SD);

# According to Geisler et al. Pteridines 2015;26:31e36.

**3–46**

**Challenge for the identification of new biomarkers for the diagnosis of Sarcopenia**

Ruben Evertz, Breno Godoy, Christine Diehl, Helge Haarmann, Amir Emami, Jochen Springer, Stephan von Haehling and Nicole Ebner

*University Medical Centre Göttingen, Cardiology and Pneumology, Göttingen, Germany*

**Background:** Sarcopenia has been observed as a Heart Failure (HF) co‐morbidity in 20 to 50% of patients, depending on the underlying aetiology and age group investigated in previous studies. The diagnosis of sarcopenia is difficult and requires the measurement of appendicular muscle mass in the DEXA (Dual‐Energy X‐Ray Absorptiometry) scan. Therefore, the identification of a biomarker to facilitate the diagnosis would be desirable. We sought to investigate the value of FGF 23 in correctly identifying sarcopenic patients with HF in a large sample of subjects from the Studies Investigating Co‐morbidities Aggravating HF (SICA‐HF).

**Methods:** A total of 198 patients with symptomatic HF were studied between Februar 2010 through June 2013 at the Charité Medical School, Campus Virchow‐Klinikum, Berlin, Germany as part of SICA‐HF Study. Patients underwent echocardiography, bicycle spiroergometry, 6 minute walk test (6 MWT), and had serum samples frozen immediately after withdrawal at −80 °C. Sarcopenia was diagnosed using the Rosetta criteria. Accordingly, sarcopenic patients were classified as those whose appendicular muscle mass (ASMI) differed by at least 2 standard deviations from a healthy comparison collective (ASMI cut off values for men 7.26, ASMI cut off values for women 5.45). All patients underwent DEXA Scan for the evaluation of the ASMI. FGF‐23 was analysed using an ELISA kit from Immundiagnostik Inc., (Bensheim, Germany) with intra‐assay varying concentration (CV) of 9.375% and inter‐assay CV of 5.254%.

**Results:** Sarcopenic patients with HF were older (72 ± 1 vs. 67 ± 1 years), had a lower Body Mass Index (25.77 ± 0.68 vs. 29.39 ± 0.39 kg/m^2^), a lower peak oxygen uptake (VO_2_ max) (13.99 ± 0.75 vs. 17.6 ± 0.42 ml/min/kg) and a lower Left Ventricular Ejection Fraction (35 vs 40%) (all *p* < 0.05). There were no differences in the 6 MWT, serum creatinine levels and New York Heart Association (NYHA) functional classification. Significant higher levels of FGF‐23 were measured in patients with sarcopenia compared to those without this condition (353.2 ± 450.8 vs. 167.7 ± 223.7 RU/mL; *p* = 0.004). FGF‐23 is a predictor of poor exercise performance in HF patients by the measured VO2 max, as well as a higher NYHA class independent of the presence of sarcopenia. FGF‐23 is not suitable for diagnosing sarcopenia in patients with HF (AUC 0.649, 95% CI 0.0553–0.746).

**Discussion:** FGF‐23 is a predictor of decreased cardiopulmonary performance in patients with heart failure, regardless of the co‐morbidity sarcopenia. FGF‐23 is a poor marker for the prediction of sarcopenia in heart failure patients. Further investigations to identify a suitable biomarker for the diagnosis of sarcopenia are required.

**3–47**

**Fibroblast Growing Factor 23 (FGF‐23) as a biomarker for the prediction of poorer exercise performance in patients with Heart Failure**

Breno Godoy, Ruben Evertz, Christine Diehl, Helge Haarmann, Amir Emami, Jochen Springer, Stephan von Haehling and Nicole Ebner

*Cardiology and Pneumology, University Medical Centre Göttingen, Göttingen, Germany*

**Background:** In patients with Chronic Heart Failure (CHF) exercise performance and NYHA (New York Heart Association) functional class are not necessarily correlated to the extent of the Left Ventricular Dysfunction (LVD). Fibroblast Growing Factor 23 (FGF‐23) has been shown to be associated with adverse cardiovascular outcomes and greater mortality in patients with Coronary Artery Disease (CAD), CHF, Chronic Kidney Disease (CKD) as well a predictor of cardiac remodeling after ST‐Segment Elevation Myocardial Infarction (STEMI). We aim to investigate the relation between this biomarker as a predictor for exercise capacity and CHF symptomatic manifestation.

**Methods:** A total of 254 patients with stable CHF were enrolled from 2010 and followed up until April 2014 or until they died. Subjects showed a mean age of 67.2 ± 10.8 years, 21.5% were female, had a Left Ventricle Ejection Fraction (LVEF) of 38.7 ± 13.3%, a body mass index (BMI) of 29.5 ± 5.5 kg/m^2^, New York Heart Association (NYHA) Class of 2.32 ± 0.65, glomerular filtration rate (GFR) calculated by CKD‐EPI of 66.5 ± 32.6 ml/min/1.73m^2^. The incidence of CAD was 55%. FGF‐23 was measured with ELISA in blood serum (162.8 ± 192.5 RU/mL). A total of 212 patients underwent cardiopulmonary exercise testing (spiroergometry) and where assigned to two groups (A: higher exercise capacity; B: lower exercise capacity) according to their maximal oxygen uptake (VO_2_ max). The median VO_2_ max of 16.45 mL/kg·min was used as cut‐off (mean 16.8 ± 9.35 mL/kg·min).

**Results:** Both groups were homogenously distributed concerning LVEF % (A: 41.1 ± 12.9 and B: 36.9 ± 13.1; *p* = 0,541), Left Atrium (LA) Diameter (A: 45.9 ± 5.9 mm and B: 49 ± 7 mm; *p* = 0.379), Left Ventricular Internal Diameter End Diastole (LVIDd) in mm (A: 56.5 ± 8.9 and B: 57.9 ± 11.3; *p* = 0.195), Interventricular Septum Diameter (IVSd) in mm (A: 12.01 ± 2.60 and B: 13.08 ± 7.86; *p* = 0.079), Lung Function defined by the Tiffeneau‐Index FVC/FEV1 (A: 76.98 ± 8.80 and B: 72.64 ± 10.20; *p* = 0.094), Heart Rate (HR) at rest in bpm (A: 63.5 ± 10.5 and B: 66.0 ± 10.2; *p* = 0.379) and Mean Arterial Pressure (MAP) in mmHg (A: 91.7 ± 13.1 and B: 94.9 ± 19.3; *p* = 0.126). There was a statistical significant difference in age distribution (A: 64.,4 ± 11.8 and B: 69.7 ± 9.3 years; *p* = 0.021), body mass index (BMI) in kg/m^2^ (A: 28.1 ± 4.3 and B: 30.2 ± 5.8; *p* < 0,001), renal function assessed by the GFR in ml/min/1,73m^2^ (A: 75.2 ± 17.4 and B: 58.6 ± 21.6; *p* = 0.006), NYHA Class (A: 1.95 ± 0.55 and B: 2.65 ± 0.55; *p* = 0.001), VO_2_ max in mL/kg·min (20.77 ± 3.03 and 12.91 ± 2.41; *p* = 0.021) and FGF‐23 in RU/mL (A: 93.5 ± 83.1 and B: 206.4 ± 219.5; *p* < 0.001). Linear Regression showed that FGF‐23 levels correlate inversely to VO_2_ max. (*R* = −0,435, p < 0.001, 95% Confidence Interval (CI) ‐0.04 to −0.22). The ROC‐Curve was used to calculate the FGF‐23 cut‐off for the prediction of lower exercise capacity with a value of 101 RU/ml (62.8% Specificity and 62.8% Sensitivity, Area Under the Curve (AUC) 0.66, *p* < 0.001).

**Discussion:** Higher Levels of FGF‐23 were positively correlated to a lower exercise capacity defined by lower VO_2_ max and are associated with a poorer functional NYHA Class independent of echocardiographic parameters for LVD (LVEF, LVIDd, IVSd and LA diameter). Patients with a poorer overall performance were also slightly older, more obese and had a lower GFR. Our findings match those with worse cardiovascular outcomes reported in the literature. However higher levels of circulating FGF‐23 are well documented in the presence of CKD, so it might be also a cofounder in patients with CHF.

**4–11**

**Gene expression and fatty acids profile in skeletal muscle of cachectic and weight stable gastrointestinal cancer patients**

Anna Taranko*^1^, Gabriela Salim de Castro^2^, Ulrike Lenz^1^, Joanna Darck Carola Correia Lima^2^, Estefanía Simões Fernández^2^, Flávio Tokeshi^3,4^, Paulo Sérgio Alcantara Martins^3,4^, José Pinhata Otoch^2,3,4^, Alison Colquhoun^2^ and Marília Cerqueira Leite Seelaender^2,3,4^

^1^ *University of Potsdam, Germany;* ^2^ *Cancer Metabolism Research Group, University of São Paulo, Brazil;* ^3^ *Department of Clinical Surgery, University Hospital, University of São Paulo, Brazil;* ^4^ *Faculdade de Medicina, University of São Paulo, Brazil*

**Introduction:** Cachexia is a wasting syndrome defined by a continuous loss of skeletal muscle mass with or without body fat loss. Muscle from cancer cachectic patients presented morphological alterations in sarcoplasmic reticulum and mitochondria, which may be related to increased oxidative stress and dysregulated energy metabolism in myocytes. Cachectic patients may present an increase in intramyocellular lipids. Myosteatosis seems to be risk factors for mortality in cancer patients. Therefore, this study aimed to characterize the fatty acid profile and mRNA expression of genes related to lipid metabolism in skeletal muscle of cachectic patients with gastrointestinal cancer.

**Methods:** Gastrointestinal cancer patients were recruited after signature of the informed consent form. Cachexia was diagnosed as in Evans 2008. *Rectus abdominis* muscle biopsies were collected in surgery. Patients were separated into Weight‐Stable Cancer (WSC) and Cachectic Cancer (CC) groups. Muscle mRNA was extracted and real time PCR gene expression was analysed using 2^‐ΔΔCT^ (WSC *n* = 17 and CC *n* = 15). Fatty acid profile was analysed by gas chromatography – mass spectrometry (WSC *n* = 8 and CC n = 8).

**Results:** Gene expression of carnitine palmitoyltransferase 1 was increased in CC patients compared to WSC patients (*p* = 0.04). No differences were observed for carnitine palmitoyltransferase 2, perilipin 2, perilipin 5 and CD36. Among muscle fatty acids, oleic acid was increased (p = 0.04) in CC patients compared to WSC patients.

**Conclusions:** These results may indicate an increased β‐oxidation in skeletal muscle of cachectic patients, and modification of fatty acid content of myocellular lipid inclusions, which may contribute to muscle wasting.

**4–12**

**Cachectic metabolome supports aggressive cancer states and survival**

David K. Thomas*, Alexander Hopkins, Nemanja Marajonavic and Todd Golub

*Cancer Program, Broad Institute of MIT & Harvard, 415 Main Street, Cambridge, MA, 02142, USA*

While it is recognized that cachexia effects profound metabolic changes in a broad range of tissues, it is poorly understood how the resultant cachectic metabolome affects the inducing cancer biology. Recent studies characterizing aggressive cancers highlight novel lipid metabolism processes as essential for sustaining aggressive features and cancer cell survival. In this study, we used IPA extraction and RP/C8 + mode MS methodology for the isolation and unbiased detection of lipid metabolites in conditioned media from differentiated human adipocytes previously exposed to cachexia‐inducing vs non‐inducing cancer cells. Of the more than 120 lipid metabolites detected, we focused on the lysophospholipids that have been implicated in aggressive cancer metabolism from other studies. Fourteen of these metabolites were detected in all samples with 5 of the 6 significantly elevated metabolites in the cachexia‐inducing exposure precisely matching the known lysophospholipids species to be taken up and utilized by *m*KRAS cancer cells. Through this focused study, we demonstrate that aggressive cancer states induce cachectic metabolic changes in primary human adipocytes, resulting in the release of specific lysophospholipid metabolites that cancer cells can uptake and utilize to support drug resistance and survival. These findings expand our understanding of fundamental cancer biology and new targetable dependencies for therapeutic development in the most challenging cancers.

**5–15**

**Myonuclear number after xenograft‐induced cancer cachexia is unchanged**

Ivan Winje^1^, Xia Sheng^1^, Kenth Arne Hansson^1^, Andreas Solbrå^1^, Jo C. Bruusgaard*^1^, Simen Tennøe^2^ and Kristian Gundersen^1^

^1^ *Department of Biosciences, University of Oslo, Oslo, Norway;* ^2^ *Department of Health Sciences, Kristiania University College, P.O. Box 1190 Sentrum, Oslo, Norway*

**Introduction:** Cachexia is a severe wasting disorder that involves severe weight loss and muscle atrophy, often resulting in immobility and cardiac/respiratory failure. In skeletal muscle, the atrophy has been reported to be accompanied by apoptosis, contributing to the atrophy. In our study, we have investigated whether myonuclei are lost during prostate cancer‐induced atrophy in skeletal muscle.

**Methods:** We induced cachexia by xenografting prostate PC‐3 cells to nu/nu mice. Six weeks after the injections, we used in vivo microscopy to measure the cross‐sectional area and myonuclei number in extensor digitorum longus muscle (EDL). Additionally, the EDL, soleus and tibialis muscles were harvested for ongoing ex vivo immunohistochemical analysis of myosin heavy chain, cross‐sectional area and myonuclei numbers. TUNEL‐labelling was performed for the presence of apoptosis in the tissue. We also investigated the general muscle condition by HE‐staining.

**Results:** Following the injections, the mice had lost 12% their peak body weight and 16% body weight compared with control animals. In vivo measurements of EDL showed a 25% decrease in myofibre CSA (*p* = 0.0022) with no apparent loss of myonuclei. This was substantiated by the finding that TUNEL‐labelling in the cachectic muscles was similar to control muscles. We also observed a decrease in heart wet weight and increased size of the spleen, accompanied by an overall 14% decrease in wet weight of the muscles analysed. HE‐sections did not differ between the groups.

**Conclusions:** Our data indicate that cancer cachexia causes no loss of myonuclei. The absence of apoptosis and abnormal HE‐staining indicate that cachexia is not a degenerative process, and the potential for recovery is latent in the muscle tissue.

**5–16**

**IL‐1 regulates metabolic dysbiosis in a chronic infection model of cachexia**

Stephanie J. Melchor, Jessica A. Hatter and Sarah E. Ewald*

*Department of Microbiology, Immunology and Cancer Biology at the Carter Immunology Center, University of Virginia School of Medicine, Charlottesville, VA, USA*

Cachexia is a leading predictor of mortality across chronic diseases. However, there are no widely efficacious interventions to reverse disease progression. As with many diseases of complex aetiology, the long‐term cachexia observed in the clinic has proven difficult to model in the lab with chemical or surgical insults. To address this need, our approach is to study the evolutionarily relevant host–parasite interaction between mice and *Toxoplasma gondii* where cachexia develops as a natural part of chronic infection. We have recently shown that adult mice infected with *Toxoplasma* loose 20% of their body mass and sustain inflammation, muscle and adipose loss over 5 months despite regaining eating. *Toxoplasma* cachexia is robust, observed across sexes, several mouse strains and animal facilities. Using a combination of host and parasite genetic tools we show that mice deficient in components of the IL‐1 pathway are protected from chronic cachexia. The liver and the brain are sites of sustained IL‐1 production and IL‐1R−/− mice have reduced inflammation in these tissues and reduced circulating IL‐6, TNF and IFN‐y. Importantly, parasite burden is unchanged in IL‐1R−/− animals, consistent with the hypothesis that IL‐1 functions as a master regulator of host homeostasis rather than as a pathogen restriction mechanism. This is consistent with the observation that cachectic mice are rely on beta‐oxidation as an energy source but this metabolic shift is rescued in IL‐1R−/− animals. Importantly, the lipid metabolism program is primarily upregulated in the liver, rather than the muscle or adipose depots. Ongoing work is aimed at pinpointing how liver‐brain cross talk regulates contributes to cachexia biology.

**5–17**

**Improved muscle fiber diameter and motoneuron number by s‐oxprenolol treatment in a mouse model of** **amyotrophic lateral sclerosis** **(ALS)**

Tsuyoshi Suzuki, Cathleen Drescher^1^, Sandra Palus^1^, Vincenzo Musolino^2^, Stephan von Haehling^1^ and Stefan D. Anker^1,3^ and Jochen Springer^1^

^1^ *Department of Cardiology & Pneumology, University Medical Center Göttingen (UMG), Göttingen, Germany;* ^2^ *Institute of Research for Food Safety & Health (IRC‐FSH), University of Catanzaro “Magna Graecia”, Catanzaro, Italy;* ^3^ *Division of Cardiology and Metabolism – Heart Failure, Cachexia & Sarcopenia, Department of Cardiology (CVK); and Berlin‐Brandenburg Center for Regenerative Therapies (BCRT); Deutsches Zentrum für Herz‐Kreislauf‐Forschung (DZHK) Berlin, Charité Universitätsmedizin Berlin, Germany*

Degeneration of upper and lower motoneurons in spinal cord, brainstem and motor cortex result in progressive neurodegenerative paralysis and death in amyotrophic lateral sclerosis (ALS), which also causes a form of cachexia. Currently, only riluzole is approved for the treatment of ALS. Here, we tested novel therapeutic options (beta blockers vs riluzole or placebo) in an internationally standardized and established model using male and female transgenic G93A ALS mice. Survival after birth was significantly improved by treatment (table 1).

**Table 1:** Hazard ratio, 95%CI and p‐value vs. placebo.

	Max. survival	Median survival	Hazard Ratio	95%CI	p‐value
Placebo	144	129.6 ± 2.1			
30 Rilutek	145	127.9 ± 2.1	1.05	0.63–1.75	0.85
10 Propoanolol	145	131.4 ± 1.6	1.10	0.63–1.91	0.74
20 Oxprenolol	149	128.1 ± 3.3	1.09	0.64–1.85	0.76
10 R‐Oxprenolol	148	133.1 ± 2.1	0.68	0.40–1.14	0.14
20 R‐Oxprenolol	146	133.7 ± 1.8	1.01	0.46–1.27	0.29
10 S‐Oxprenolol	157	138.1 ± 1.5** ^### $^	0.42	0.26–0.69	0.005
20 S‐Oxprenolol	166	139.0 ± 2.0** ^### §^	0.53	0.31–0.91	0.020

** : *p* < 0.01 vs placebo,

### : *p* < 0.001 vs rilutek,

$ : *p* < 0.05 vs respective doses of r‐oxprenolol.

In a second set of experiments, effects of compounds were tested on body weight, biochemical parameter, myocyte diameter and motoneuron number 41 days after first symptoms of ALS (Table 2).

**Table 2:** LBM: Lean body mass, Mstn: Myostatin aktive (25 kDa) and pro‐form (52 kDa), Chymo: Chymotrypsin‐like, PGPH: PGPH‐like, und Tryp: Trypsin‐like proteasome activity in nmol/mg protein/ min, myofiber: Myofiber diameter (M. tibialis).

	Placebo	30 Rilutek	20 R‐Oxpren	10 S‐Oxpren	20 S‐Oxpren
Weight loss g	−1.96 ± 0.23	−1.34 ± 0.23	−1.56 ± 0.36	−0.91 ± 0.38*	−1.13 ± 0.36*
loss LBM g	−1.74 ± 0.30	−0.99 ± 0.37	−1.54 ± 0.42	−0.50 ± 0.41*	−0.61 ± 0.38*
Mstn 25 kDa AU	1.13 ± 0.09	0.99 ± 0.11	0.84 ± 0.08	0.74 ± 0.07*	0.76 ± 0.11*
Mstn 52 kDa AU	0.54 ± 0.06	0.54 ± 0.08	0.40 ± 0.07	0.38 ± 0.05	0.32 ± 0.03*
chymo	1633 ± 138	1527 ± 206	1891 ± 167	1307 ± 174	1330107
PGPH	1096 ± 146	1127 ± 140	1177 ± 103	860 ± 126	973 ± 61
tryp	1400 ± 166	1101 ± 117	1381 ± 122	924 ± 140*	973 ± 83*
myofiber μm	35.24 ± 1.09	34.74 ± 1.80	38.19 ± 2.11	39.61 ± 2.14	41.55 ± 1.76*, *
cortex	336 ± 43	482 ± 27*	465 ± 25*	525 ± 32*, *	483 ± 25*
Spinal cord	327 ± 30	474 ± 48*	497 ± 45*, *	475 ± 43*	656 ± 46*, *

* : p < 0.05,

** : *p* < 0.01 vs Placebo.

In summary, *S*‐oxprenolol improves survival by protecting mononeurons, reducing loss of body weigt and lean mass via downregulation of wasting related signaling.

**6–11**

**Skeletal muscle mitochondrial energy metabolism in cancer cachexia: Clinical and mechanistic approaches**

Adeline Dolly*, Julie Cournet, Jean‐François Dumas and Stéphane Servais

*INSERM UMR1069, “Nutrition, Croissance et Cancer”, University of François Rabelais, Tours, France*

**Introduction:** Cancer‐associated cachexia is a wasting disorder caused by negative energy balance, which dramatically diminishes quality of life and survival. One important feature of cachexia is the progressive loss of skeletal muscle mass. Despite growing interest and knowledge, there are still unexplored mechanistic pathways underlying cancer cachexia. Evidence from pre‐clinical models of cancer cachexia and key works on muscle cells suggest proteolytic pathways, mitochondrial dysfunctions and sarcoplasmic reticulum stress to be important factors that could contribute to the development of skeletal muscle atrophy associated with cancer cachexia. However, no clinical studies on muscular mitochondrial bioenergetics have been performed yet.

**Methods:** A clinical pilot study (METERMUCADIG, NCT02573974) is including 45 patients with pancreatic or colorectal cancer. Pectoral muscle biopsies and blood samples are collected before chemotherapy. Patients muscle mass is assessed by CT‐Scan at the third lumbar vertebrae.

We also use an *in vitro* model of mouse skeletal muscle cells, C2C12 for the mechanistic analyses of muscle atrophy.

**Results:** Our preliminary data on muscle biopsies seem to show a decrease of mitochondrial oxygen consumption linked to ATP synthesis (35%), energy wasting (30%) and maximal respiratory capacity (25%) in cachectic patients in comparison to non‐cachectic patients. This suggests a global reduction in mitochondrial bioenergetics in cachexia that can be explained by specific respiratory complex alteration and/or decrease in mitochondrial content.

*In vitro*, we show that TNFα, a proinflammatory cytokine, induces a dose‐dependent atrophy of differentiated C2C12 myotubes. Treating C2C12 with 125 ng/mL of TNFα for 48 h resulted in a significant myotube atrophy by 32%, without affecting mortality.

**Conclusions:** Our data in skeletal muscle of cachectic patients seem to confirm mitochondrial bioenergetics alterations as described in murine models. Furthermore, our *in vitro* model of muscle atrophy will permit us to determine the links between muscle atrophy, mitochondrial energy metabolism and sarcoplasmic reticulum stress.

**6–12**

**The habenula as a novel link between the homeostatic and hedonic pathways in cancer‐associated weight loss: a pilot study**

Maria Maldonado^†, 1^, David L. Molfese^†, 2,4^, Jose M. Garcia*^†, 1,3^ and Ramiro Salas^†, 2,4^

^1^ *Division of Endocrinology, Diabetes and Metabolism, MCL, Center for Translational Research on Inflammatory Diseases, Dan L. Duncan Cancer Center, Michael E. DeBakey Veterans Affairs Medical Center, Dept. of Medicine, Baylor College of Medicine, Houston, TX, USA;* ^2^ *Menninger Department of Psychiatry and Behavioral Sciences, Baylor College of Medicine, Houston, TX, USA;* ^3^ *Geriatric Research, Education and Clinical Center(GRECC), VA Puget Sound Health Care System, and Dept. of Medicine, Div. of Gerontology & Geriatric Medicine, Univ. of Washington School of Medicine, Seattle, WA, USA;* ^4^ *Michael E. DeBakey Veterans Affairs Medical Center, Houston, TX, USA*

^†^These authors contributed equally to this work

**Introduction:** Little is known about the brain mechanisms underlying cancer‐associated weight loss (C‐WL) in humans despite this condition negatively affecting their quality of life and survival. We tested the hypothesis that C‐WL patients have abnormal connectivity in homeostatic and hedonic brain pathways together with altered brain activity during food reward.

**Methods:** In 12 cancer patients and 12 healthy controls, resting state functional connectivity (RSFC, resting brain activity observed through changes in blood flow in the brain which creates a blood‐oxygen‐level dependent [BOLD] signal that can be measured using functional magnetic resonance imaging[fMRI]) was used to compare three brain regions hypothesized to play a role in C‐WL: the hypothalamus (homeostatic), the nucleus accumbens (hedonic), and the habenula (an important regulator of reward). In addition, the brain reward response to sweet juice was studied.

Participants included twelve patients with histological diagnosis of incurable cancer (solid tumors), a European Cooperative Oncology Group (ECOG) performance status of 0–2, and a ≥ 5% involuntary body weight loss from pre‐illness over the previous 6 months and 12 non‐cancer controls matched for age, sex, and race. RSFC between the hypothalamus, nucleus accumbens, and habenula, and brain striatum activity as measured by functional MRI during sweet juice reward delivery events were the main outcome measures.

**Results:** After adjusting for BMI and compared to matched controls, C‐WL patients were found to have reduced RSFC between the habenula and hypothalamus (*p* = 0.04) and between the habenula and nucleus accumbens (*p* = 0.014). C‐WL patients also had reduced reward responses in the striatum compared to controls.

**Conclusions:** In C‐WL patients, reduced connectivity between both homeostatic and hedonic brain regions and the habenula, and reduced reward to sweetness were observed. The habenula and striatum are potential therapeutic targets in C‐WL.

**Acknowledgements:** This study was funded by a MEDVAMC Seed grant (JG, RS), VHA MERIT grants (I01BX002807 to JG, CX000174 to JG, and CX000994 to RS), the NCI (3P30CA125123‐08S2 to RS) and the NIA T32AG000183 and AG040583 to JG. Funding was also provided by the American Federation of Aging Research via the Medical Student Training in Aging Research program to MM, and the McNair Medical Institute to RS.

**6-13**

**Role of myostatin, activin A and follistatin in cachexia of ENT cancers**

Alexias Dissard*^1^, Yves Boirie^1^, Stéphane Walrand^1^, Nicolas Saroul^2^ and Laurent Gilain^2^

^1^ *Human Nutrition Department, ENT Department, Clermont‐Ferrand, France;* ^2^ *ENT Department, Clermont‐Ferrand Hospital*

**Introduction:** Myostatin and activin A, two members of the superfamily TGF‐β, have been shown to play a role on skeletal muscle mass regulation. In Humans, high plasma concentrations of activin A were observed in cancer patients, especially in cachectic subpopulations. The main objective of our study was to determine the modifications of blood myostatin, activin A and follistatin concentrations associated with head and neck cancers.

**Methods:** 55 patients were included in the study: 32 in the cancer group (only squamous cell carcinoma) and 23 in the control group. The patients underwent a complete nutritional assessment and multiple samples: blood before and 7 days after surgery, skeletal muscle biopsies, tumour biopsies. Plasma concentrations of myostatin, activin and follistatin were measured before and after tumour removal surgery. Concentrations of myostatin, activin and follistatin were also measured in an incubation medium of a tumour biopsy.

**Results:** Activin A and follistatin plasma concentrations were significantly increased in the cancer group (320 vs. 203 pg/ml; *p* < 0.001) (3593 vs 2148 pg/ml; p < 0.001), while myostatin plasma concentration was significantly decreased in this group (1542 vs. 2100 pg/ml; *p* = 0.010). Surprisingly, data of the 7th postoperative day showed an increase in plasma activin A concentration (379 vs 320 pg /ml; p < 0.001) while concentrations of myostatin and follistatin were not modified. A high postoperative systemic inflammation could explain these results. Myostatin, activin A and follistatin proteins were systematically detected in the medium of tumour a 48 hour‐incubation period, providing a strong proof of the tumour production of these factors by squamous cell carcinoma.

**Conclusions:** The activin A/myostatin/follistatin is modified in the context of head and neck cancer. Activin A particularly seems to play a role in the occurrence of cachexia while follistatin could have a protective role for skeletal muscle mass.

**6-17**

**The impact of race/ethnicity on the prognostic associations between general and visceral obesity and breast cancer outcomes**

Shalini Dalal*, David Hui, Brinda Rao Korivi, Diane Liu, Shiva Dibaj, Rony Dev, Nikhil Sobti, Eduardo Bruera and Jennifer Litton

*Department of Palliative Care and Rehabilitation Medicine, The University of Texas M. D. Anderson Cancer Center, 1515 Holcombe Boulevard, Unit 1414, Houston, TX, 77030, USA*

**Introduction:** Racial/ethnic differences in breast cancer (BC) survival is well recognized. Factors such as higher stage at diagnosis, unfavorable tumour characteristics, treatment received and obesity may play a role and merit further research. This retrospective study aimed to investigate the differences in the associations between obesity (BMI) especially visceral obesity (high visceral‐adipose tissue [VAT] accumulation) on BC prognosis by race/ethnicity.

**Methods:** VAT was measured from computed tomography imaging. Associations between BMI, VAT (high and low), clinico‐pathological factors and outcomes were examined.

**Results:** Among 1154 patients, 67% were white, 17% black and 16% Hispanic. Ninety one percent of black patients were either overweight or obese (25% and 66%, respectively), as were 86% Hispanics (37% and 49%), compared to 72% whites (32% and 40%, *P* < .001). Among obese patients, a lower proportion of black (27%) as compared to Hispanic (57%) and white (56%) patients had high VAT (*p* < .001). A higher proportion of black patients had higher nuclear grade (p < .001), stage (*p* = .036) and triple‐negative BC (*p* < .001) as compared to white and Hispanic patients, and a higher proportion of black and white patients had lymphovascular invasion as compared to Hispanic patients (*p* < .001). No differences in type/duration of neoadjuvant‐chemotherapy were observed (*p* < 0.5). In multivariate‐adjusted analyses, high VAT was associated with lower chance of pathological complete response (OR = 0.55, 95% CI 0.32 to 0.96) and higher risk for recurrence (HR = 1.91, 95% CI 1.30 to 2.79) and death (HR 2.04, 95% CI 1.33 to 3.12) only among white patients. Patients with normal‐BMI as compared to obese patients had higher risk of death (HR = 1.77, 95% CI 1.12 to 2.79) only in white women.

**Conclusions:** Our study demonstrates racial/ethnic differences in the associations between obesity and BC outcomes, with high VAT and low BMI being independent prognostic for poorer outcomes only in white patients.

**6-18**

**Intractable nausea and anorexia with weight loss in a patient with advanced breast cancer**

Ronny Dev*

*University of Texas MD Anderson Cancer Center, Houston, TX, USA*

**Aim:** Highlight benefits of olanzapine for the control of nausea, anorexia resulting in weight loss in a patient with advanced breast cancer being evaluating in an outpatient Anorexia‐Cancer Cachexia Clinic.

**Case Presentation:** A 72 year old female with metastatic breast cancer involving the bilateral breasts, sternum and left proximal femur, who had initially undergone a right‐sided mastectomy and subsequently had recurrence noted in left breast, sternum and left proximal femur which was receiving active treatment with letrozole, palboiciclib and denosumab at an outside institution. She presents for consultation with intractable nausea (rated 10/10) with occasional vomiting, anorexia (rated 7/10) with 35 pound weight loss. Nausea was triggered by overeating, movement and associated with diaphoresis above the neck. Previous work‐up included unremarkable upper and lower endoscopy and normal computed tomography scan of abdomen and gastro‐motility studies. Patient's nausea was extensively treated with ondansetron, promethazine, meclizine, and amitriptyline without benefits. Subsequently, she was treated with keppra followed by topiramate for possible migraines induced nausea by a neurologist, which were ineffective in controlling symptoms. Patient denied rumination or anxiety triggering nausea but acknowledged that her quality of life had declined due to her symptom burden. For symptomatic treatment of nausea, patient was prescribed olanzapine 2.5 mg by mouth every 12 hours as needed. She was also advised to follow‐up with neurologist and recommended weaning off topiramate which had been ineffective. At 1 and 3 month follow‐up, patient rating of nausea was 0/10 without anorexia and was gaining weight while tolerating treatment.

**Conclusions:** Olanzapine, an atypical antipsychotic, has potent anti‐nausea effects via blocking multiple neuronal receptors. Olanzapine has potent 5‐HT3 inhibitory effects with a longer half‐life than ondansetron as well as antianxiety effects. Olanzapine should be considered for chemotherapy‐related and advanced cancer‐related nausea and more research is needed with double‐blind studies comparing with other class of anti‐emetics.

**6-19**

**Serum exosomal MicroRNA‐21 could revolutionise the early molecular diagnosis of cancer cachexia**

Ayman Aboda*^1^, Wafaa Taha^2^, Iman Abdelgawad^2^, Ahmed Gad^2^, Mahmoud Mohsen^3^ and Jagat Rakesh Kanwar^1^

^1^ *Nanomedicine‐Laboratory of Immunology and Molecular Biomedical Research (NLIMBR), Centre for Molecular and Medical Research (C‐MMR), Deakin University, 75 Pigdons Road, Waurn Ponds, VIC, 3220, Australia;* ^2^ *National Cancer Institute, Cairo University, Fom El Khalig, Cairo, 11796, Egypt;* ^3^ *Faculty of Pharmacy, Cairo University, Egypt*

**Background:** Advanced approach for the diagnosis of cancer cachexia is crucially required. There is developing enthusiasm in employing microRNAs (miRNAs) as biomarkers in diseases. Exosomes are vesicles with a diameter between 40‐nm to 100‐nm and are produced by a range of cells and are important for cell–cell communication. This study aims to determine if the miRNA in exosomes from sera of cancer cachexia patients could be validated as a biomarker for early diagnosis of cancer cachexia.

**Methods:** Exosomes were isolated from the sera of patients with cachexia linked to non‐small cell lung cancer or ovarian cancer and patients without cachexia, but with the same cancer types. Total RNA was purified from the exosomes with analysis of expression levels of microRNA‐21 by quantitative real‐time polymerase chain reaction (RT‐PCR).

**Results:** Exosomal expression levels of micRNA‐21 were notably higher in cancer cachexia patients compared to those without cachexia, but the same cancer type. Over expression of exosomal miRNA‐21 was significantly correlated with both tumour staging and clinical classification of the tumour.

**Conclusions:** Exosomal micRNA‐21 should be utilised as a biomarker for the early diagnosis of cancer cachexia at pre‐cachexia stage as it is validated in this study with a significant number of participants. Exosomal micRNA‐21 was positively correlated with percent of decrease in BMI and tumour staging**,** indicating it could be a potential target for cancer cachexia therapy.

**6-20**

**High‐dose espindolol prevented muscle wasting and improved survival in a rat model of severe cancer cachexia**

Junichi Ishida*, Masakazu Saitoh, Nicole Ebner, Stefan D. Anker and Jochen Springer

*Innovative Clinical Trials, Department of Cardiology and Pneumology, University Medical Centre Göttingen, Göttingen, Germany*

**Background and aims:** Cancer cachexia is a multifactorial disorder, characterized by progressive weight loss and anorexia. Skeletal muscle wasting is associated with poor prognosis in this condition and enhanced chronic adrenergic stimulation could partially contribute to this pathological setting. In this study, we tested the hypothesis that beta‐adrenergic blockade prevents loss of body weight and skeletal muscle, resulting in better survival in a rat cachexia model.

**Methods:** 10^8^ Yoshida AH‐130 hepatoma tumour cells were intraperitoneally injected into rats and they were randomized to receive placebo, espindolol 0.3 mg/kg/day (LD) or 3 mg/kg/day (HD). On day 16 or earlier due to reaching ethical endpoints, body weight and body composition were measured, and samples obtained from gastrocnemius muscle were evaluated by western blotting analysis.

**Results:** HD preserved both body weight (219.2 ± 17.3 g for HD, *p* < 0.01, 182.6 ± 13.1 g for LD, *p* = 0.05 vs. 152.4 ± 1.9 g for placebo) and lean body mass compared to placebo (170.0 ± 13.5 g for HD, *p* < 0.001, 145.7 ± 10.4 g for LD, p < 0.01 vs. 118.5 ± 1.6 g for placebo), resulting in prolonged survival (HR, 0.29; 95% CI, 0.16‐0.51, p < 0.001 for HD; HR, 0.51; 95% CI, 0.26‐1.00, *p* = 0.051 for LD, vs placebo). Protein analysis showed that cancer cachexia significantly decreased expression of beta‐2 adrenergic receptor in gastrocnemius muscle by 0.30 times compared to control rats, but that HD reversed this effect and increased protein levels related to protein synthesis such as phosphorylated phosphoinositide 3‐kinase and protein kinase B.

**Conclusions:** High‐dose espindolol could prevent muscle wasting and weight loss through maintained beta‐2 adrenergic signaling, resulting in better survival in cancer cachexia.

**6-21**

***Drosophila* as a model for cancer cachexia**

Rojyar Khezri^1,2^, Nadja Katheder^1,2^, Ashish Jain^1,2^ and Tor Erik Rusten^1,2^

^1^ *Department of Molecular Cell Biology, Institute for Cancer Research, Oslo University Hospital, MontebelloN‐0379, Oslo, Norway;* ^2^ *Centre for Cancer Biomedicine, Faculty of Medicine, University of Oslo, MontebelloN‐0379, Oslo, Norway*

**Background and aims:** Cancer cachexia is a devastating systemic tissue wasting syndrome elicited by tumour presence in up to 80% of late stage patients and estimated to account for 20% of cancer‐related deaths. This starvation‐like response occurs despite sufficient feeding and is characterized by insulin resistance, metabolic changes, fatigue, and gradual degradation of carbon and lipid sources, in particular from muscle and adipose tissue. The cellular response in target tissues is a switch from an anabolic to catabolic state. The systemic responses of cancer cachexia are believed to be in part a consequence of inflammatory signaling initiated by and from the tumour, but mechanistic studies in *in vivo* model systems has been limited. Our aim is to establish a genetic model where we can mechanistically dissect the mechanisms of cachexia, and if cachectic wasting contributes to tumour growth and nutrient remobilization fueling tumour growth.

**Methods:** The animal model system, *Drosophila melanogaster*, is a powerful model to study molecular, genetic and cellular mechanisms of cancer *in vivo*. In this system, genetically defined organ‐specific tumors can be produced, and metabolic changes and somatic cellular responses to tumour presence can be studied in target muscle and adipose tissues.

**Results:** Using transgenic organ‐specific markers and dyes, we have been able to identify and quantify organ size of muscle and adipose tissue, as well as tumour growth over time by microscopy. Our studies have shown that *Ras* ^*v12*^ *;scrib* tumors but not *Ras* ^*v12*^ *;control* tumors cause cachexia in the distal tissues: muscle and fat body.

**Conclusions:** *Drosophila melanogaster* can be used as a model for cancer cachexia. This model will be used to study the moleculargenetic mechanism of cachexia.

**7-21**

**Dietary protein intake and lean mass in patients with lung or colorectal cancer**

Ilana Roitman*^1,2^, Chantelle A. Benson^1^, Melissa A. Bontorin^1^, Vickie E. Baracos^3^ and Carla M. Prado^1^

^1^ *Human Nutrition Research Unit, Department of Agricultural, Food and Nutritional Sciences, University of Alberta, Canada;* ^2^ *Cancer Institute of Sao Paulo, Faculty of Medicine Foundation of the University of Sao Paulo, Brazil;* ^3^ *Department of Oncology, University of Alberta, Canada*

**Introduction**: Low lean mass (LM) is a common issue among cancer patients and can negatively affect their prognosis^1^. Although an adequate supply of protein is essential to sustain LM, protein intake of patients recently diagnosed with cancer has not been extensively examined. Therefore, our objective was to evaluate the amount and type of protein consumed and to explore the association between LM and protein intake in patients with advanced solid tumors.

**Methods:** Patients were recruited from the Cross Cancer Institute (Edmonton, Alberta). Dietary intake was evaluated using a three‐day food record and analysed for protein amount and source. Protein intake was compared to the Recommended Dietary Allowance (RDA, 0.8 g/kg/day) and the body weight (BW) adjusted European Society for Clinical Nutrition and Metabolism (ESPEN, minimum 1.0 g/kgBW/day, target of 1.2‐2 g/kgBW/day) protein guidelines. Protein intake was divided into animal and plant sources. Body composition was measured by dual energy x‐ray absorptiometry (DXA).

**Results:** In this preliminary analysis, a total of 51 patients with metastatic lung (*N* = 24) or colorectal cancer (*N* = 27) were included. No differences in protein intake were observed between cancer groups. Mean protein intake for all patients was 90 ± 24 g/day, and 1.15 ± 0.32 g/kg BW/day; 90% met the RDA and 39% met ESPEN guidelines target protein intake of ≥1.2 g/kgBW/d. Only 1 patient consumed ≥2.0 g/kgBW/day. Animal sources accounted for 62% of total protein intake. A wide variation was observed in protein intake per unit LM with a weak relationship between these variables (r^2^ = 0.208, *p* < 0.001).

**Conclusions:** Less than 40% of patients met the target protein recommendation for cancer reflecting the need for additional nutrition support/intervention as low protein intake could potentially increase the risk for low lean mass or sarcopenia. As LM drives protein requirements, understanding the protein intake profile of cancer patients may enable the development of protein recommendations and future targeted interventions.

**7-22**

**Oligonol, a low‐molecular weight polyphenol, alleviates aging related sarcopenia in senescence‐accelerated mice**

Wei‐Tai Tseng*, Yun‐Ching Chang, Xin‐Ling Wu and Sue‐Joan Chang*

*National Cheng Kung University, Tainan, Taiwan*

Sarcopenia is an aging‐related disease with a significant reduction in mass and strength of skeletal muscle due to the imbalance between protein synthesis and protein degradation. Oligonol, a low molecular weight polyphenol derived from lychee, exhibited anti‐diabetic and anti‐obesity properties, suggesting being a proper supplement for attenuating age‐related muscle loss. Sarcopenia was investigated in senescence‐accelerated mice supplemented without and with Oligonol (200 mg/kg) for 8 weeks. Oligonol supplementation significantly increased muscle mass, muscle strength and fiber cross‐sectional areas by up‐regulating protein synthesis via phosphorylation of mTOR and P70^S6K^ in SAMP8 mice. In addition, Oligonol prevented nuclear translocation of NFκB and FoxO1a, thereby suppressing Atrogin‐1 and MuRF1 expression. Oligonol reduced inflammatory mediators and inhibited autophagy by decreasing the levels of LC3II/LC3I. In conclusions, our results suggest oligonol as a supplement for alleviating sarcopenia evidenced by improving protein synthesis via mTOR and P70^S6K^ and suppressing protein degradation via NFκB and FoxO1a in SAMP8 mice.

**7-23**

**Effect of medical cannabis on appetite and weight: A retrospective analysis**

Maria Fernanda Arboleda and Vi Dam and Youri Drozd and Erin Prosk and Popi Kasvis and Michael Dworkind and Robert Kilgour and Antonio Vigano*

**Introduction:** The orexigenic effect of cannabinoids has been extensively studied in patients with cancer and HIV/AIDS.^1^, ^2^ However, there is little published evidence of the orexigenic effects of synthetic (e.g., dronabinol) and natural cannabinoids (e.g., delta‐9‐tetrahydrocannabinol (Δ9‐THC) and/or cannabidiol (CBD)).^3^, ^4^, ^5^ This retrospective study aimed to explore the effect of medical cannabis on appetite and weight.

**Methods:** We conducted a retrospective chart review of all adult patients who were assessed at Santé Cannabis Clinic between August 2016 and July 2017, and who had “increase of appetite” as a treatment goal. Santé Cannabis Clinic is the only medical cannabinoid clinic located in the province of Quebec, Canada. Patient characteristics, “lack of appetite” item (0 = no lack of appetite to 10 = complete lack of appetite) from the revised Edmonton Symptom Assessment System (r‐ESAS), and weight (kg) were considered our primary outcomes.

**Results:** A total of one hundred and eleven patients were identified as suffering from anorexia from different chronic conditions, but only forty patients had complete information available at baseline and at three months follow‐up. The r‐ESAS score for lack of appetite significantly improved by 2.3 ± 3.4 points (*p* < 0.05 by Wilcoxon rank test); no statistically significant change in the mean weights (baseline vs three month; 74.7 ± 22.7 kg vs 75.1 ± 23.6 kg; *p* = 0.121) was found in our sample. The most common route of administration was a combination of oral and inhaled medical cannabis (50%). Forty percent of the patients received THC‐rich products. Overall, 27.5% of the subjects reported only mild side effects (i.e., not impacting function nor requiring discontinuation of cannabis) such as anxiety, fatigue, dizziness and dry mouth.

**Conclusions:** Chronic administration of medical cannabis may safely improve appetite and help with weight maintenance in patients suffering anorexia from cancer as well as non‐malignant chronic diseases.

**References:**

**7–24**

**The green tea polyphenol epigallocatechin‐3‐gallate attenuates skeletal muscle atrophy in a senescence‐accelerated mouse model of sarcopenia**

Xin‐Ling Wu*, Yun‐Ching Chang and Wei‐Tai Tseng and Sue‐Joan Chang

*National Cheng Kung University, Tainan, Taiwan*

Senescence‐accelerated mouse prone 8 (SAMP8) shortens the process of aging and may facilitate an alternative model for studying aging‐related sarcopenia. In present study, molecular mechanism of aging‐related skeletal muscle atrophy in SAMP8 model of sarcopenia established by our laboratory at aged 3, 6, 8, and 10 months was investigated. Results showed that the peaks of muscle mass and muscle strength were observed at aged 8 months and the declines of that were observed at aged 10 months. Therefore, 8‐month‐old SAMP8 were fed with control diet or control plus 0.32% of EGCG diet for 8 weeks. SAMP8 supplemented with EGCG had significantly greater gastrocnemius muscle mass, fiber cross‐sectional areas and muscle strength than that of control without EGCG supplementation. EGCG normalized AKT activity, resulting in reduced FoxO1a expression in the nuclear fraction, leading to decreased expression of Atrogin‐1 and MuRF1 in SAMP8 mice compared with age‐matched SAMR1. EGCG also increased protein synthesis via mTOR/p70sk6 signaling pathway. In addition, EGCG ameliorated mitochondrial dynamics fusion and fission, which contribute to the inhibiting autophagy evidenced by decreased Atg13 and LC3II/LC3I levels during the aging. In conclusions, EGCG supplementation preserves muscle by attenuating protein degradation via ubiquitin‐proteasome and the autophagy‐lysosome systems and promoting protein synthesis via mTOR/p70^sk6^ pathway in sarcopenia mice.

**8–21**

**The effects of C26 induced cancer cachexia and activin receptor ligand blocking on skeletal muscle metabolome**

Juulia H. Lautaoja*^1^, Maciej Lalowski^2^, Tuuli A. Nissinen^1^, Jaakko Hentilä^1^, Sulin Cheng^3,4^, Olli Ritvos^5^ and Juha J. Hulmi^1,5^

^1^ *Biology of Physical Activity, Neuromuscular Research Center, Faculty of Sport and Health Sciences, University of Jyväskylä, Jyväskylä, Finland;* ^2^ *Medicum, HiLife, Biochemistry/Developmental Biology, Meilahti Clinical Proteomics Core Facility, University of Helsinki, Helsinki, Finland;* ^3^ *Faculty of Sport and Health Sciences, University of Jyväskylä, Jyväskylä, Finland;* ^4^ *Exercise, Health and Technology center, Shanghai Jiao Tong University, Shanghai;* ^5^ *Department of Physiology, Faculty of Medicine, University of Helsinki, Helsinki, Finland*

**Background and aims.** Blocking of the activin receptor ligands using a soluble form of the activin receptor type IIB (sACVR2B‐Fc) was shown to prevent cachexia and improve survival in C26 cancer when treated throughout the experimental cancer (Nissinen et al. SCWD Berlin 2016) but the mechanisms are unknown. The aim of this follow‐up study was investigate skeletal muscle metabolome of cachectic mice with or without activin receptor administration.

**Methods.** BALB/c male mice were randomized into 4 groups: 1) healthy controls (CTRL) with placebo treatment (PBS) and C26 tumour‐bearing mice treated with 2) placebo (C26 + PBS), 3) sACVR2B‐Fc treatment only before C26 tumour formation (C26 + sACVb), and 4) sACVR2B‐Fc treatment both before and after the C26 tumour formation (C26 + sACVc). Gastrocnemius muscles were collected on day 11 after C26 cell inoculation when the loss of body weight most successfully predicted survival in our previous survival experiment. Metabolites were analysed by gas chromatography–mass spectrometry (GC–MS), and further bioinformatic searches were performed with Ingenuity software. Statistical significance was set at *P* < 0.05 and fold change >1.3.

**Results.** In the cachectic C26 muscle the most altered metabolites and pathways were related to protein synthesis and amino acid metabolism. These effects were in part attenuated by continued activin receptor ligand blocking (C26 + sACVc). Of the single metabolites other than amino acids, beta‐glycerophosphoric acid and iminodiacetic acid were among the most altered ones in cancer while glucose‐6‐phosphate and L‐aspartic acid were the most affected by activin receptor ligand blocking.

**Conclusions.** Skeletal muscle metabolome is altered in C26 cancer cachexia and this is affected, in part, by the blocking of activin receptor ligands. The role and significance of the altered metabolites will be investigated in further detail in the future.

*This work was supported by Academy of Finland grant No. 275922*.

**8–22**

**Activin receptor ligand blocking in tumour‐bearing mice: effects on acute phase response and spleen expansion**

Tuuli A. Nissinen*^1^, Jaakko Hentilä^1^, Fabio Penna^2,3^, Juulia H. Lautaoja^1^, Anita Lampinen^1^, Arja Pasternack^4^, Olli Ritvos^4^, Riikka Kivelä^5^ and Juha J. Hulmi^1,4^

^1^ *Biology of Physical Activity, Neuromuscular Research Center, Faculty of Sport and Health Sciences, University of Jyväskylä, Jyväskylä, Finland;* ^2^ *Department of Clinical and Biological Sciences, University of Turin, Turin, Italy;* ^3^ *Interuniversity Institute of Myology, Italy;* ^4^ *Department of Physiology, Faculty of Medicine, University of Helsinki, Helsinki, Finland;* ^5^ *Translational Cancer Biology Research Program and Wihuri Research Institute, University of Helsinki, Helsinki, Finland*

**Background and aims.** Cancer‐induced acute phase response (APR) and expansion of myeloid‐derived suppressor cells (MDSCs) have been linked to reduced survival in cancer cachexia. In addition, we (Nissinen et al. SCWD Berlin 2016) and others have found that blockade of activin receptor type IIB (ACVR2B) ligands is associated with improved survival in cachectic mice. The aim of this study was to investigate the effects of C26 tumour and ACVR2B ligand blocking on liver proteins/APR and splenic MDSC content.

**Methods.** BALB/c mice were injected with vehicle (control) or C26 cancer cells and treated with PBS or soluble ACVR2B (sACVR2B‐Fc, 5 mg/kg twice a week, three injections before and three injections after C26 cell injection). Spleen and liver samples were collected on days 11–13 after C26 cell injection. Statistical significance was set at *P* < 0.05.

**Results.** C26‐induced muscle wasting was blocked by sACVR2B‐Fc. Liver mass was unaltered in the experimental groups, but liver protein synthesis was increased in PBS‐treated tumour‐bearing mice, while sACVR2B‐Fc attenuated this effect. Increased liver protein synthesis was associated with higher phosphorylation of Stat3 and accumulation of fibrinogen and serpinA3N, known markers of APR, in tumour‐bearing mice. APR was attenuated in some but not all sACVR2B‐Fc treated mice. Spleen mass was increased in tumour‐bearing mice and this effect was partly blocked by sACVR2B‐Fc. Preliminary transcript and immunofluorescence analyses suggest an increase in splenic MDSCs in tumour‐bearing mice without attenuation by sACVR2B‐Fc administration.

**Conclusions.** These results suggest that blocking ACVR2B ligands improves survival independently from splenic MDSC content. By contrast, prevention of cachexia by sACVR2B‐Fc might be associated with attenuated liver APR. Further investigation is required in order to clarify the importance of this finding with respect to survival.

*This work was supported by Academy of Finland grant No. 275922, Jenny and Antti Wihuri Foundation and the Finnish Concordia Fund*.

**9–04**

**Targeted medical nutrition for cachexia in NSCLC: a randomized, controlled trial**

Alessandro Laviano^1^, Philip C. Calder^2,3^, Annemie Schols^4^, Fredrik Lonnqvist^5^, Maria Öhlander^6^ and Maurizio Muscaritoli*^1^

^1^ *Department of Clinical Medicine, Sapienza University of Rome, Rome, Italy;* ^2^ *Human Development and Health Academic Unit, Faculty of Medicine, University of Southampton, Southampton, UK;* ^3^ *NIHR Southampton Biomedical Research Centre, University Hospital Southampton NHS Foundation Trust and University of Southampton, Southampton, UK;* ^4^ *Department of Respiratory Medicine, NUTRIM School of Nutrition and Translational Research in Metabolism, Maastricht University Medical Centre, Maastricht, Netherlands;* ^5^ *Department of Molecular Medicine and Surgery and the Center for Molecular Medicine, Karolinska Institute, Stockholm, Sweden;* ^6^ *Smartfish AS, Oslo, Norway*

**Background:** Cachexia is a complex metabolic syndrome associated with an accelerated decline in physical function, impaired health‐related quality of life (HRQoL) and premature mortality in patients with non‐small‐cell lung cancer (NSCLC). This double‐blind, placebo‐controlled, randomized trial evaluated the safety and efficacy of targeted medical nutrition (TMN) in (pre‐)cachectic patients with NSCLC.

**Methods**: Patients receiving first‐line chemotherapy for NSCLC with involuntary weight loss or low BMI were randomized 1:1 to receive TMN (~200 kcal; 10 g whey protein; > 2.0 g eicosapentaenoic acid−/docosahexaenoic acid‐containing fish oil; and 10 μg 25‐hydroxy‐vitamin D3) or a milk‐based isocaloric comparator (~200 kcal; sunflower oil in place of fish oil; no vitamin D3) twice daily for 12 weeks (http://clinicaltrials.gov Identifier: NCT02515032). Primary safety endpoints included number and type of adverse events and changes in vital signs and laboratory parameters. Secondary efficacy endpoints included changes in body weight, body composition, exercise capacity, inflammatory and metabolic biomarkers and HRQoL. Chemotherapy‐related outcomes were exploratory endpoints. Statistical analyses were performed for the full analysis set and a predefined per protocol set (study completers with >70% compliance).

**Results**: Fifty‐five patients were randomized to receive TMN (*n* = 26; mean 64.4 ± 7.7 years) or isocaloric comparator (*n* = 29; mean 66.0 ± 8.0 years). TMN was well tolerated; the TMN group experienced fewer adverse events (64 vs 87), including fewer cases of neutropenia (0 vs 4), than the comparator group. Heart rate decreased from baseline in the TMN group and increased in the comparator group in highly compliant patients at week 12 (*p* < 0.05). Triglyceride levels also decreased in the TMN group and increased in the comparator group in the per protocol patients (−0.28 mmol/L vs +0.43 mmol/L; *p* = 0.0027). Body weight increased to a similar extent in both groups and body composition remained similar. The omega‐3:omega‐6 ratio and vitamin D3 levels were higher in the TMN versus the comparator group at study end (both *p* < 0.0002). There were no other significant differences between the groups, including in chemotherapy‐related outcomes of tumour size, dose‐limiting toxicity and dose reductions.

**Conclusions:** TMN is well tolerated in (pre‐)cachectic patients with NSCLC. Both study groups gained weight, and TMN had an additional positive impact on vital signs and lipid profiles. In addition, this study showed signs of improved chemotherapy tolerability in the TMN group. Thus, TMN could be beneficial for the nutritional support of lung cancer patients receiving chemotherapy.

**9–05**

**Bovine Lactoferrin for treatment of anaemia associated with cachexia, pilot study for a randomised controlled trial**

Ayman Aboda*^1^, Wafaa Taha^2^, Iman Abdelgawad^2^ and Jagat Rakesh Kanwar^1^

^1^ *Nanomedicine‐Laboratory of Immunology and Molecular Biomedical Research (NLIMBR), Centre for Molecular and Medical Research (C‐MMR), Deakin University, 75 Pigdons Road, Waurn Ponds, VIC, 3220, Australia;* ^2^ *National Cancer Institute, Cairo University, Fom El Khalig, Cairo, 11796, Egypt*

**Background:** Cachexia is defined as loss of muscle due to serious sickness such as chronic heart failure, cancer, chronic renal failure and chronic obstructive pulmonary disease. Lactoferrin is well known as multifunctional protein, bovine Lactoferrin has several biological function, such as its effect in improvement of immunity, antimicrobial and anticancer effects.

**Method/Design:** A prospective randomized controlled trail (pilot study) was conducted on 40 participants (30 patients with cancer cachexia and 10 participants apparent healthy) to check the effect of bovine Lactoferrin to improve anaemia in cancer cachexia patients with underlying stage IV non‐small cell lung cancer and in healthy participants.

**Results:** Both groups were matched regarding age, and sex. After 12 weeks, there was significant difference in haemoglobin concentration, serum iron level and red blood cells count in participants who received 250 mg/day bovine Lactoferrin in comparison to those who received placebo as P value was <0.001. Also there was significant difference in haemoglobin concentration between those who received 250 mg bovine Lactoferrin in comparison to those received 5oo mg bovine Lactoferrin as P value was <0.003.

**Conclusions:** Oral bovine Lactoferrin should be included in the multimodal therapy for treatment of cancer cachexia.

**9–06**

**Investigating the safety and impact on muscle mitochondria of orally administered Urolithin A: a randomized, double‐blind, placebo controlled Phase 1 clinical trial in elderly**

Anurag Singh*^1^, Pénélope A. Andreux^1,2^, William Blanco‐Bose^1^, Patrick Aebischer^3^, Johan Auwerx^2^ and Chris Rinsch^1^

^1^ *Amazentis SA (EPFL Innovation Park, Batiment CCH‐1015, Lausanne, Switzerland;* ^2^ *Laboratory for Integrative and Systems Physiology School of Life Sciences, Ecole Polytechnique Fédérale de Lausanne, CH‐1015, Lausanne, Switzerland;* ^3^ *Neurodegenerative studies laboratory Brain Mind Institute, School of Life Sciences, Ecole Polytechnique Fédérale de Lausanne, CH‐1015, Lausanne, Switzerland*

**Background:** Age associated muscle and physical decline leading to frailty and sarcopenia has become a growing public health concern. This has led to the search for novel nutritional interventions that can delay or mitigate its progression. Urolithin A (UA), is a first‐in‐class food derived metabolite that has recently been shown to improve mitochondrial function and exercise capacity in animal models of age‐related decline of muscle function (*Nature Medicine* 22, 879–888 (2016). The current study was designed to investigate the safety and impact on mitochondrial biomarkers with an orally administered, synthetically produced UA in a Phase 1 clinical trial in elderly.

**Methods:** The safety and tolerability of UA and it's impact on muscle mitochondrial biomarkers was evaluated in a single‐center, double‐blind, randomized study (NCT02655393). This was a 2‐part study with a single oral ascending dose Part A and a 4 week multiple ascending dose Part B. In Part A, 24 healthy elderly male and female volunteers [12 females and 12 males, ranging in age from 61 to 82 years (mean 68.7 ± 5.3 years) and body mass index (BMI) ranging from 20.2 to 30.4 kg/m^2^ (mean 24.60 ± 2.72 kg/m^2^)] were randomized (6 subjects/group) to consume UA in single ascending doses of either 250 mg, 500 mg, 1,000 mg and 2,000 mg or placebo. Each dose was separated by a washout period of 3 weeks. Similarly, in Part B, the safety and tolerability of UA was evaluated following a 28‐day (4 week) oral administration. 36 healthy elderly male and female volunteers [12 males and 24 females, ranging in age from 61 to 78 years (mean age 66.4 ± 4.9 years) and BMI ranging from 18.8 to 30.6 kg/m^2^ (mean of 25.02 ± 3.04 kg/m^2^)] were randomized (9 subjects/group) to receive 250 mg, 500 mg, or 1,000 mg of UA or placebo daily for 28 days. In Part A and Part B, UA was administered orally, in fasting condition, in soft‐gel formulations with water for all dosing's. Additionally, in Part A, UA was administered in high protein yogurt at doses of 500 mg and 1,000 mg. In each study, subjects underwent physical examinations and electrocardiogram (ECG) evaluations and were monitored for adverse events. Liver and kidney function [*i.e*., creatinine, uric acid, alanine serine transferase (AST), alanine leucine transferase (ALT), gamma glutamyl transferase (GGT), and total and conjugated bilirubin] were evaluated before and after dosing's. Full laboratory tests included hematology [*i.e*., hemoglobin, hematocrit, red blood cells (RBC), white blood cells (WBC), differential count, platelet count, mean corpuscular volume (MCV), mean corpuscular hemoglobin (MCH), mean corpuscular hemoglobin concentration (MCHC)] and urinalysis (pH, ketone bodies, proteins, glucose, and blood). Plasma and muscle biopsies from the *vastus lateralis* muscle were collected to investigate the effects of UA on the skeletal muscle transcriptome and on the plasma metabolomics profile in elderly.

**Results:** In the single dose Part A phase there were no serious adverse events (SAE) recorded for any dosing. 5 of 24 subjects reported the occurrence of 6 non‐serious adverse events, of which none were considered related to intake of UA. Adverse events were distributed across all dose groups, including placebo. No clinically significant abnormal laboratory test values from study baseline were observed for any of the biochemistry tests assessing liver and kidney function, or for any of the hematology and urinalysis tests for any subjects at any of the doses during the course of the study. No abnormal and clinically significant conclusions were observed for ECG findings for any subjects taking active intervention at any of the doses during the course of the study. As a result, it was concluded that single dosing of UA at the doses of 250 mg, 500 mg, 1,000 mg, or 2,000 mg was safe and well tolerated. In the multiple‐dose (28 day/oral intake) Part B phase, no serious adverse events were reported. 31 non‐serious adverse events were reported in 15 subjects (the majority being linked to study procedures, i.e. muscle biopsy), none were considered to be related to intake of UA. No clinically significant changes were reported in liver and kidney function tests, hematology or urinalysis. No clinically relevant and abnormal findings were reported during physical examination. Vital signs were likewise unaffected. No significant abnormalities were reported during ECG examinations. UA oral administration significantly up‐regulated mitochondrial gene expression in skeletal muscle and lowered plasma acylcarnitines.

**Conclusions:** UA is well tolerated and has a favourable safety profile when orally administered in single and multiple doses to elderly. UA significantly modulated mitochondrial biomarkers in elderly. Our results hold promise for the use of UA to manage age‐associated declines in muscle and mitochondrial function.

**10–14**

**Minimally supervised home‐based resistance training programs and muscle function of older and middle‐aged adults: A systematic review and meta‐analysis**

Ofer Kis^1,2^, Assaf Buch*^2,3,4^, Limor Ben‐Haim*^2^, Naftali Stern^2,3^ and Daniel S. Moran^1^

^1^ *The Faculty of Health Sciences, Ariel University, Ariel, Israel;* ^2^ *Institute of Endocrinology, Metabolism and Hypertension, Tel Aviv Sourasky Medical Center, Tel‐Aviv, Israel;* ^3^ *The Sackler Faculty of Medicine Tel‐Aviv University, Israel;* ^4^ *Robert H Smith Faculty of Agriculture, Food and Environment, The Hebrew University of Jerusalem, Rehovot, Israel*

**Background:** Despite the proven effectiveness of resistance training in older adults, low participation rates continue to pose a major problem in attempts to retard age‐related sarcopenia, frailty, and functional limitations. Home‐based resistance training (HBRT) has been increasingly prescribed to remedy this situation.

**Objective:** To evaluate the efficacy and safety of minimally supervised HBRT on muscle strength and functionality in older populations, including those afflicted with chronic diseases. Design: a systematic review and meta‐analysis. Data sources: MEDLINE (Ovid), EMBASE and Cochrane electronic databases were systematically searched including references lists. Eligibility criteria: randomized controlled studies comparing HBRT to controls (637 articles identified) with an outcome measure of lower and/ or upper body strength and/ timed up and go test (TUG) (Figure 1). Two independent reviewers appraised methodological quality. Sensitivity analysis was performed with the inclusion of studies which presented relatively lower risk of bias.

**Results:** seventeen studies were included (15 in the meta‐analysis) with a total of 1754 subjects (70% females), aged 69.18 ± 9.82 yrs. Lower and upper body strength significantly increased by 0.77 kg (95% CI, 0.32–1); SMD 0.26 (95% CI, 0.07–0.45) (Figure 2). TUG improved by 0.73 sec’ (95% CI, −1.38, −0.08) (figure 3). Subgroup analysis revealed enhanced effects of exercise intensity, duration, volume and contact level on lower body strength. High contact level and program duration were associated with increases in upper body strength. Exercise intensity improved TUG test.

**Conclusions:** minimally supervised HBRT increases lower and upper body strength and functionality in diverse populations of older and middle‐aged adults.

**10–15**

**There are no no‐responders to low or high resistance training volumes among older women**

Paulo Gentil*^1^, Mathues Barbalho^2^, Mikel Izquierdo^3^, James Fisher^4^, James Steele^4^ and Rodolfo Raiol^5^

^1^ *Universidade Federal de Goiás, Goiânia, Brazil;* ^2^ *Centro de Ciências Biológicas e da Saúde, Universidade da Amazônia, Belém, PA, Brazil;* ^3^ *Department of Health Sciences, Public University of Navarre, CIBER de Fragilidad y Envejecimiento Saludable (CB16/10/00315), Tudela, Navarre, Spain;* ^4^ *School of Sport, Health, and Social Sciences, Southampton Solent University, United Kingdom;* ^5^ *Programa de Pós‐Graduação em Fisioterapia, Centro Universitário do Estado do Pará, Belém, PA, Brazil*

**Introduction:** One aspect that is frequently debated regarding prescription of resistance training (RT) is exercise volume, reflected in the number of sets performed. Moreover, there is wide interindividual variability in the results obtained from RT and some subjects can be classified as responders (R) no non‐responders (NR). The present study aimed to assess the prevalence of NR to different tests and to compare the effects of different RT in older women.

**Methods:** 376 women performed 12 weeks of RT with either low (LV) or high volume (HV). Both groups performed the same exercises, and all parameters were held constant except for the number of sets performed per week. LV performed 8–12 for upper and 4–6 for lower body, while HV performed 16–20 and 8–10, respectively. Before and after the training period, the participants were tested for bench press and leg press 1RM, 30‐second chair stand, 30‐seconds arm curl, six‐minute walk test, sit and reach, body weight and waist circumference.

**Results:** both groups significantly improved in all strength and functional tests and reduced their body weight and waist circumference. ANOVA revealed higher gains in the leg press 1RM, 30‐seconds arm curls and 6‐minute walk test for the HV group and higher increases in the results of the sit and reach test for the LV group. However, the differences were negligible and may be attributable to a type I error due to the large sample size. Non‐responsiveness was not apparent in any subject, as a positive response on at least one outcome was present in every participant.

**Conclusions:** Our results suggest that RT, even at low volume, improves waist circumference, muscle strength and physical function in the older population, with no evidence of non‐responsiveness. Therefore, we should not be restrictive in prescribing this type of exercise to this population.

**10–16**

**Differences in balance ability according to leg preference in the female elderly**

Ye‐ji Jung*^1^, Yun‐jeong Baek^1^ and Chung‐hwi Yi^2^

^1^ *Dept. of Physical Therapy, The Graduate School, Yonsei University, South Korea;* ^2^ *Dept. of Physical Therapy, College of Health Science, Yonsei University, South Korea*

**Introduction:** People preferentially use one side of the body to perform voluntary motor action. This tendency characterizes the lateral preference. This leg dominance resulting from lateral preference has been indicated as a potential contributing factor to interlateral balance asymmetries, dynamic stability asymmetries, and subsequent performance asymmetries. The purposes of this study were to identify differences in balance ability between dominant leg and non‐dominant leg in the female elderly.

**Methods:** The female elderly over 65 years old were recruited (*n* = 16). They performed one leg standing (OLS) on dominant leg and non‐dominant leg alternatively under three sensory conditions (eyes open/firm ground, eyes close/firm ground, eyes open/foam ground) in random order. During OLS, we collected data of balance using a six‐component force plate. Two‐way repeated measures analysis of variance was used for statistical analysis, and the Bonferroni correction was used as a post hoc test.

**Results:** There were significant main effects of leg for area of center of pressure (COP) (*p* = .046) and significant main effects of condition for all analysed balance measure including COP displacement in antero‐posterior (AP) and medio‐lateral (ML) directions, COP velocity in AP and ML directions, resultant velocity of COP, total length of COP, and area of COP (*p* < .001).

**Conclusions:** These findings suggest that although differences between legs nearly were not statistically significant different, with increasing level of sensory condition (eye open/firm ground < eye open/foam ground < eye close/firm ground), postural sway significantly increased in female elderly.

**10–17**

**The cost‐effectiveness of an exercise‐based comprehensive intervention in older adults**

Yuya Watanabe*^1,2,3^, Tsukasa Yoshida^4,5^, Miwa Yamaguchi^4^, Yosuke Yamada^4,3^ and Misaka Kimura^2,3^

^1^ *Faculty of Health and Sports Science, Doshisha University;* ^2^ *Faculty of Health and Medical Sciences, Kyotogakuen University;* ^3^ *Laboratory of Sports and Health Science, Kyoto Prefectural University of Medicine;* ^4^ *Department of Nutrition and Metabolism, National Institutes of Biomedical Innovation, Health and Nutrition;* ^5^ *Department of Senior Citizen's Welfare, Kameoka‐City*

**Introduction**: Japan as a super aged society faces problems related to the lifestyle and health of older people. Costs of healthcare such as medical and long‐term care insurance (LTCI) service are expected to swell with the future increase in the older population. In Japan, exercise‐based intervention for the older people is extensively conducted to improve or maintain their physical function. Although such programs are considered effective to prevent sarcopenia and/or frailty, their cost‐effectiveness is controversial. Therefore, we aimed to clarify the cost‐effectiveness of an exercise‐based comprehensive intervention.

**Methods**: The program consisted of exercise combined with oral function care and nutritional guidance. We provided the program to 523 older adults. Meanwhile, 523 controls were matched using the propensity score calculated from the items of the Survey of Needs in the Spheres of Daily Life carried out before the intervention. We tracked LTCI requirement certifications and death for 5 years from July 2011 and investigated the incidence of events in both groups. We also compared LTCI service charge and medical cost (National Health Insurance) in the observation period.

**Results**: Sixty nine (13.3%) in the intervention group and 98 (19.2%) in the control group were newly certified to be eligible for LTCI service with a significant difference (*p* = 0.012). The odds ratio of LTCI certification/death in the control group was 1.40 (95% CI, 1.07–1.82). The total LTCI service cost was 57,419,158 yen and 130,370,021 yen in the intervention and control groups, respectively, with a difference of 72,950,863 yen. However, no significant difference was observed in mortality or medical cost between the groups.

**Conclusions**: Our study, which showed that an exercise‐based comprehensive intervention suppressed the number of new LTCI certifications and LTCI service cost and, thus, saved the health‐care cost by about 73 million yen, provided positive evidence of cost‐effectiveness of such an intervention.

**10–18**

**A pilot study for randomized controlled trial of the benefits of a physical exercise program for cancer cachexia patients**

Ayman Aboda*^1^, Wafaa Taha^2^, Adel Elkady^3^ and Jagat Rakesh Kanwar^1^

^1^ *Nanomedicine‐Laboratory of Immunology and Molecular Biomedical Research (NLIMBR), Centre for Molecular and Medical Research (C‐MMR), Deakin University, 75 Pigdons Road, Waurn Ponds, VIC, 3220, Australia;* ^2^ *National Cancer Institute, Cairo University, Fom El Khalig, Cairo, 11796, Egypt;* ^3^ *Police Force Hospital, Giza, Egypt*

**Background:** Cachexia is a wasting disorder that has been associated with a range of illness, including cancer. For instance, non‐small cell lung cancer (NSCLC) has quite a high incidence of cachexia (34%). Cachexia is a cause of morbidity and mortality. Physical exercise programme may improve physical performance and quality of life among cancer cachexia patients.

**Method/Design:** A prospective two‐armed randomized, controlled (RCT) pilot study was conducted on thirty participants (15 cases and 15 controls) to check the effects of a physical exercise program (12 weeks) compared to usual care on physical performance and quality of life in cancer cachexia patients with underlying stage IV non‐small cell lung cancer.

**Results:** Both groups were matched regarding age, sex and diagnosis. There was significant improvement in physical performance among the case group who received the intervention program for 12 weeks which is manifested by significant improvement in the walking distance in 6‐minute walk test, P value was <0.001, significant improvement in hang grip strength test P value was <0.001. There was also significant improvement in both the short physical performance battery test, P value was <0.001 and in the quality of life among the case group, P value was <0.001. Moreover, there was a significant improvement in depression, which is manifested by a decrease in the Center for Epidemiologic Studies Depression Scale (CES‐D‐20) score.

**Conclusions:** A Physical exercise program was found to be clinically and statistically significant as the patients gain improvement in the physical performance, quality of life and depression, so it should be considered essential part of the multimodal therapy for cancer cachexia patients. This may help them to cultivate independency as long as possible with better quality of life in their survival period.

**10–19**

**Early signaling events involved in muscle remodeling after exercise**

Francesca Solagna^1,2^, Leonardo Nogara^1,2^, Kenneth Dyar^3^, Franziska Greulich^3^, Henriette Uhlenhaut^3^, Irene Moretti^1,2^, Kristian Vissing^4^, Marcus Krüger^5^ and Bert Blaauw*^1,2^

^1^ *University of Padova, Italy;* ^2^ *Venetian Institute of Molecular Medicine, Italy;* ^3^ *Helmholtz‐Zentrum München, Germany;* ^4^ *Aarhus University, Denmark;* ^5^ *University of Cologne, Germany*

Muscle plasticity after exercise is achieved by different molecular mechanisms that regulate gene transcription by impinging on chromatin structure and transcription factors. We performed an unbiased quantitative phosphoproteomics approach in vivo in order to determine the early signaling changes occurring after high‐intensity exercise. We identified two histone modifications which are strongly increased after exercise and potentially linked to increased gene transcription. CHIP analysis confirmed that H3S10 phosphorylation happens on SRF‐dependent genes like Fos, Jun and Egr1. Furthermore, we identify a completely new phosphorylation of MRTF‐B, a transcriptional co‐activator of SRF‐dependent gene transcription, which is critical for its nuclear localization. Based on the results achieved so far, we are proposing a model in which histone modifications and nuclear translocation of MRTF‐B act in concert for the activation of SRF‐dependent gene transcription, a well‐known mediator of exercise‐induced muscle remodeling. Identification of these early remodelling processes will be critical in identifying the proper exercise protocol leading to improved muscle performance, particularly relevant in fragile, cachectic subjects.
